# Graph neural networks can predict ketosynthase substrate specificity

**DOI:** 10.1038/s41598-026-47323-x

**Published:** 2026-05-09

**Authors:** Maxim Walmsley, Jack A. Connolly, Eriko Takano, Rainer Breitling

**Affiliations:** Manchester Institute of Biotechnology, Department of Chemistry, School of Natural Sciences, Faculty of Science and Engineering, University of Manchester, Manchester, UK

**Keywords:** Biochemistry, Chemistry, Computational biology and bioinformatics, Drug discovery

## Abstract

Modular polyketide synthases are a class of biosynthetic machinery responsible for the production of a chemically diverse range of natural products, including essential medicines. The modular architecture of this system presents as inherently rearrangeable; however, manipulation of these pathways to produce novel polyketides often results in a reduced output or the pathway stalling entirely. One cause of such breakdown is the ability of ketosynthases (KS), an essential component of the biosynthetic modules, to proofread the substrate produced by preceding steps and reject unexpected chemistries. Here we explore the KS proofreading phenomenon using Graph Neural Networks (GNN) trained on KS AlphaFold structures, as a means to predict when proofreading occurs and how to mitigate it. Our GNN models can distinguish six out of ten β-carbon reduction state pairwise combinations (81–92% AUC). The α-methylation state on the polyketide substrate can also be partially predicted from the KS structure (79% AUC). To explore the utility of the GNNs as a co-pilot in mitigating proofreading, we ran a proof-of-concept experiment, where we show that the GNN models can be used to predict beneficial mutations to a KS receiving a non-native substrate. We also present a pilot study on acyltransferase domains, which identifies novel regions potentially associated with substrate specificity.

**Supplementary Information:**

The online version contains supplementary material available at 10.1038/s41598-026-47323-x.

## Introduction

Modular polyketide synthases (mPKSs) are a class of biosynthetic machinery that produce a variety of essential polyketide natural product medicines and their semi-synthetic derivatives^1^, and notably include several antibiotics^2–5^, anticancer drugs^6^ and immunosuppressants^7^. The chemical structures produced by mPKSs are often highly complex^1^ and are consequently challenging to reproduce using synthetic chemistry in an economical manner^8^. Modification to these biosynthetic pathways in vivo is therefore appealing, both to increase the output of fermentative processes that provide us with existing essential medicines, and also to unlock new-to-nature medicinal compounds that could hold improved or novel pharmacological profiles.

The architecture of an mPKS pathway is comprised of a repeating set of modular structures that form an assembly line, where each module carries out an extension and variable modification step in the elongation of polyketide carbon chain, in a collinear manner^9,10^. Modules are comprised of three essential domains: a ketosynthase (KS), an acyltransferase (AT) and an acyl carrier protein (ACP). The AT domain imports short carbon chains used to extend the polyketide chain, the ACP moves the polyketide between domains within a module and to neighbouring modules, and the KS performs a decarboxylative Claisen condensation reaction between the polyketide output of the previous module with the extension chain, outputting a longer polyketide chain^9,11,12^. The condensation reaction produces a ketone group on the β-carbon of the polyketide, which can be modified to an alcohol group if a ketoreductase (KR) is present, an alkene if a KR and dehydratase (DH) are present, or to a methylene if a KR, DH and enoyl-reductase (ER) are present in the module. Configuration of alcohols and alkenes are predominantly determined by the KR, where KR types a and b produce l and d configured alcohols, and *cis* and *trans* alkenes respectively. The output polyketide of an mPKS pathway is therefore intrinsically coupled to the number, position and domain composition of modules in the pathway. Pathways can be further categorised into *trans*-AT or *cis*-AT, depending on whether the AT domain is incorporated into the mPKS polypeptide^13^– here, our focus is on *cis*-AT pathways.

Over evolutionary time scales, modules can be modified, duplicated, rearranged or deleted in mPKSs^14,15^, demonstrating that they can operate as discrete, interchangeable components of a pathway, and that this can be used to drive chemical diversification. We can exploit this inherent malleability through modification of mPKS genes to create novel products^16–18^. However, such modifications are often met with substantially reduced productivity or the pathway stalling entirely^16,17,19–21^.

Several explanations as to why modified pathways break down have been previously proposed and explored in the literature^20,22,23^. One explanation is the phenomenon of KS proofreading, where KSs exhibit a degree of substrate specificity towards the incoming polyketide chain^18,21,23,24^. Analysis of how modules rearrange over evolutionary periods has suggested that KS domains associate most strongly with the AT, ACP and reductive domains immediately preceding it, and not those following^15,22^. This would place the KS in a position where it can ensure that the extension and reductive steps within its discrete evolutionary unit have been correctly performed. By gating against incomplete or incorrect reactions, KSs within a pathway can collectively enforce fidelity in the output polyketide. However, this specificity can cause the pathway to break or slowdown when modification to the pathway introduces chemistries that the KS is proofreading against.

Contemporary work has experimentally identified residues in the KS that relate to substrate specificity through targeted mutagenesis^23–25^. *Trans*-AT mPKSs have KSs that form phylogenetic clusters based on polyketide substrate, and this relationship to substrate specificity has been demonstrated through mutagenesis experiments^26,27^. This phylogenetic relationship is not immediately apparent in *cis*-AT mPKSs, which instead form clusters based on pathway, rather than substrate^23,28^(though comparison of KSs within pathways have been reported to cluster on substrate^15^). Sequence analysis of *cis*-AT mPKSs has identified some associations between residues in the active site pocket of KSs and substrate chemistry^15,29^. We build on this body of literature by examining the relationship between 3D structure and substrate specificity, by developing a machine learning pipeline that can identify subtle structural differences that correlate with enzymatic function, and suggest actionable targets for mutagenesis. We propose that the methodology developed here would also be broadly applicable to enzyme design and engineering tasks beyond mPKS pathways.

In this study, we generated 995 KS AlphaFold structures as training data for a series of binary classification Graph Neural Networks (GNNs) to predict properties of native substrates. We found that binary classifiers trained on KS structures can reliably discriminate between a 6 out of 10 of the pairwise tested β-carbon reduction states: ketone, l and d alcohol, alkene (combined *cis* and *trans*), and methylene. We were unable to train models that discriminate β-methylenes from β-ketones or β-alcohols of both l and d orientations. We were also unable to train a model to distinguish l-type β-alcohols from β-ketones. We found that KS structures can be used to predict whether a 2*S*-Methylmalonyl-CoA (Mmal) or a Malonyl-CoA (Mal) extension unit is used in the preceding KS’s condensation reaction (79% AUC), a proxy for a-carbon methylation, but were unable to predict the substrate supplied to the subject KS’s extension reaction. This provides evidence towards a KS-last definition of module boundaries. To explore the validity of our GNN approach and aid in method development, we ran a pilot study on AT domain substrate specificity, which we also report here.

Using a machine learning model explainer, we produce heat maps of residues that are frequently detected as influential on the model’s predictions. Some of these residues have already been validated experimentally^23,24^, whilst others are novel and warrant experimental validation. Building from the heat maps and a trained GNN, we run a proof-of-concept experiment where we partially rescue the effect of inferred proofreading brought about by an ER knockout substrate change in the erythromycin mPKS, 6-deoxyerythronolide B synthase (DEBS), achieving a near 2-fold increase in product. The limited scale of this pilot does not provide comprehensive, mechanistic descriptions of KS proofreading; instead, it demonstrates a new means for tackling this phenomenon in a mechanism-agnostic manner, and for hypothesis generation for directed mutagenesis more broadly.

## Methods

Code, AlphaFold structures and substrate labels produced for this study have been made open access on GitHub: https://github.com/mwalm/ketosynthases.

### Protein sequence curation

We used the ClusterCAD^30^ database as a curated source of polyketide Biosynthetic Gene Clusters (BGCs) and intermediate compounds. BGC accession numbers were used to acquire antiSMASH^31,32^ annotation files from MIBiG^33^ and protein sequences for mPKS enzymatic domains were parsed from these files. From this, we produced databases of 971 AT domain and 1138 KS domains, and their associated substrates. BGCs and distribution of substrates can be found in the supplementary material (Figures S1–5).

### Structure prediction

KS and AT protein sequences were as described in the antiSMASH BGC files, which uses InterPro^34^ definitions of KS and AT domains^32^. Sequences were converted to FASTA file format and passed to ColabFold^35^ version 1.5.1 (a modified AlphaFold 2.0 pipeline^36,37^). using default settings, to generate structure predictions. KS structures were generated as homodimers and AT domains as monomers. Top ranked predictions were extracted and aligned against an arbitrarily chosen structure.

### Graph neural network training and architecture

For binary classification tasks, we used balanced category datasets, with structures sampled randomly, to a limit defined by the minority class. The balanced dataset was split, with 20% of structures being partitioned to test data – the purpose of the test partition is to monitor model performance by using data unseen by the model. Protein structures were converted from PDB files to graph network format using Graphein^38^ version 1.4, using the K-nearest neighbour construction method (k = 4). Graph networks were constructed at α-carbon resolution. Amino acid types were used as node features via a one-hot encoding method. Network graphs were imported to PyTorch Geometric^39^ and trained on a 2-layer Graph Convolutional Neural Network (GCN) using the Kipf and Welling 2016 algorithm^40^, followed by a pooling layer and linear activation layer (Figure S6, supplement). Data was run in batches of 64 with a learning rate of 0.001. Both GCN layers used a neural density of 64 using rectified linear unit functions. We used the ADAM optimiser and Cross Entropy Loss function to direct gradient descent. Structures from the erythromycin pathway were not included in the KS classifiers training data – this was to prevent structure-specific feature recall in the mutagenesis experiment.

In summary, these networks treat a protein structure as a graph where each amino acid is a node connected to its four nearest amino acid neighbours. The GCN layers update the information represented on each amino acid by combining it with that of its neighbours, forming a description of the local structural environment. The neurons in these layers act as filters that respond when they detect a learned pattern, and these filters are applied across all nodes, creating updated representations for each node. This process of neighbourhood aggregation occurs twice, so individual nodes will encounter descriptions of their 2nd degree neighbours in the second GCN layer. The resulting node representations are pooled to a single protein-level representation, which is passed through a final layer to predict the class.

### Interpretation of models

To generate heat maps of regions and residues that influence model predictions, we used GNNExplainer^41^. This identifies nodes and subgraphs that contribute to a model’s assigned substrate label. The analysis was performed on all structures in the test data partition for a given model and significant nodes were extracted on a per-structure basis. Using a defined reference structure, test data α-carbons coordinates were aligned to the reference to create a node-to-sequence mapping. This mapping was used to calculate the frequency with which a residue position was identified by GNNExplainer, represented as a percentage.

For interpretation of the heat maps, the k-nearest neighbour graph construction and two GCN layers creates an approximate radius of 5Å and 10Å information sharing on given residue’s node. Consequently, a residue may be identified as important due to its own identity, its first or second order neighbours and their residue types, or a combination of these factors. Importantly, the heat maps show the frequency of detection, not the magnitude of effect on the prediction. These maps therefore indicate regions and residues where sequence or structural variation is likely relevant to classification.

### 2.5Å proximity search and logo diagrams

AlphaFold structures were divided by class and aligned to chosen template structures using Prody^42^ 2.5.0. The template structure was used to perform a pairwise alignment of α-carbons. The α-carbons coordinates in the template sequence are used to search for α-carbons in the compared sequences that fall within a 2.5Å radius of the template α-carbons. Where multiple α-carbons were detected, the closest atomic coordinates were used. Where no residues are detected, a gap token is recorded. The relative proportions of amino acid type and gaps are calculated as a percentage, rounded to the nearest percent. The proportions were then used to generate logo diagrams using ggseqlogo^43^, using the bits format (Shannon entropy^43,44^.

### DEBS ER knockout and rescue plasmids

DEBS *E. coli* expression vectors pBP130 and pBP144, as described in Pfeifer et al. 2001^45^, were acquired from Kerafast, Boston. We replicated the ER NADPH binding domain knockout mutation (GGVGMA➔SPVGMA) described by Donadio et al. 1993^46^, in plasmid pBP130, via a three part Gibson assembly^47^. The pBP130-ER-KO plasmid was subsequently modified to incorporate a *lacZa* “socket” counter selection DNA section (supplied as synthetic DNA, Twist Bioscience) that replaced the KS sequence in module 5 of DEBS. This socket was removed and replaced by modified KS parts via a three part Gibson assembly. Plasmids were verified by restriction digest and Sanger sequencing.

### Fermentation of ER4 knockout products

RQ5 *E. coli*^48,49^ was first transformed with pBP144 via electroporation, then, in a second round, with the pBP130-ER4-KO derivative plasmids. Single colonies were picked and inoculated to 10 ml Lysogeny Broth (Miller), supplemented with kanamycin (50 µg ml^− 1^) and carbenicillin (100 µg ml^− 1^), and grown overnight at 37 °C, 180 rpm. 100 µl was used to inoculate 10 ml growth media (5 g l^− 1^ yeast extract, 10 g l^− 1^ casein extract, 15 g l^− 1^ glycerol, 10 g l^− 1^ sodium chloride, pH 7.6) supplemented with kanamycin and carbenicillin in 50 ml tubes. Cultures were grown at 37 °C, 180 rpm, to OD_600_ = 1.1. 100 µl 2 M sodium propionate and 10 µl 0.1 M IPTG was added to the cultures and were transferred to a 22 °C incubator, 240 rpm, and grown for 42 h. 1-ml samples were taken for extraction.

### Extraction and mass spectrometry

Culture samples were centrifuged at 13,000 g for 1 min. Supernatant was removed by pipetting. Pellets were discarded. Supernatant was mixed 1:1 with ethyl acetate, vortexed, and then centrifuged at 13,000 g for 1 min. 900 µl ethyl acetate layer was transferred to fresh tubes and evaporated using a GeneVac EZ-2 centrifugal evaporator. The residue was resuspended in 450 µl HPLC-grade methanol. Methanol extracts were syringe-filtered using a 0.22 μm PVDF membrane. Extracted samples were run on a Thermo Fisher, Q-Exactive orbitrap mass spectrometer in positive ionisation mode, using a Hypersil Gold C18 HPLC column (Thermo Fisher) and a 12-minute, 0–90% methanol elution. Extracted ion counts (EIC) were produced using a custom script with a 2 ppm mass range for the hydrogen adduct of compound **1** (mass range: 385.2577–385.2593. Compound structure in Fig. 8).

## Results

### Structure predictions in ColabFold

AT domain structures were predicted with uniform predicted Local Distance Difference Test (pLDDT) scores above 90% (Figure S7, supplementary). KS dimer structures were not predicted with uniform high accuracy. The core structure around the active site is confidently predicted (> 90% pLDDT). However, approximately 12 residues are consistently predicted in the 50–70% pLDDT range (see supplementary Figure S8). Scores in this range indicate confidence over the position of alpha carbons but not R-groups.

### Acyltransferases pilot experiment – assessing GNN applicability to substrate specificity tasks

**Fig. 1:**
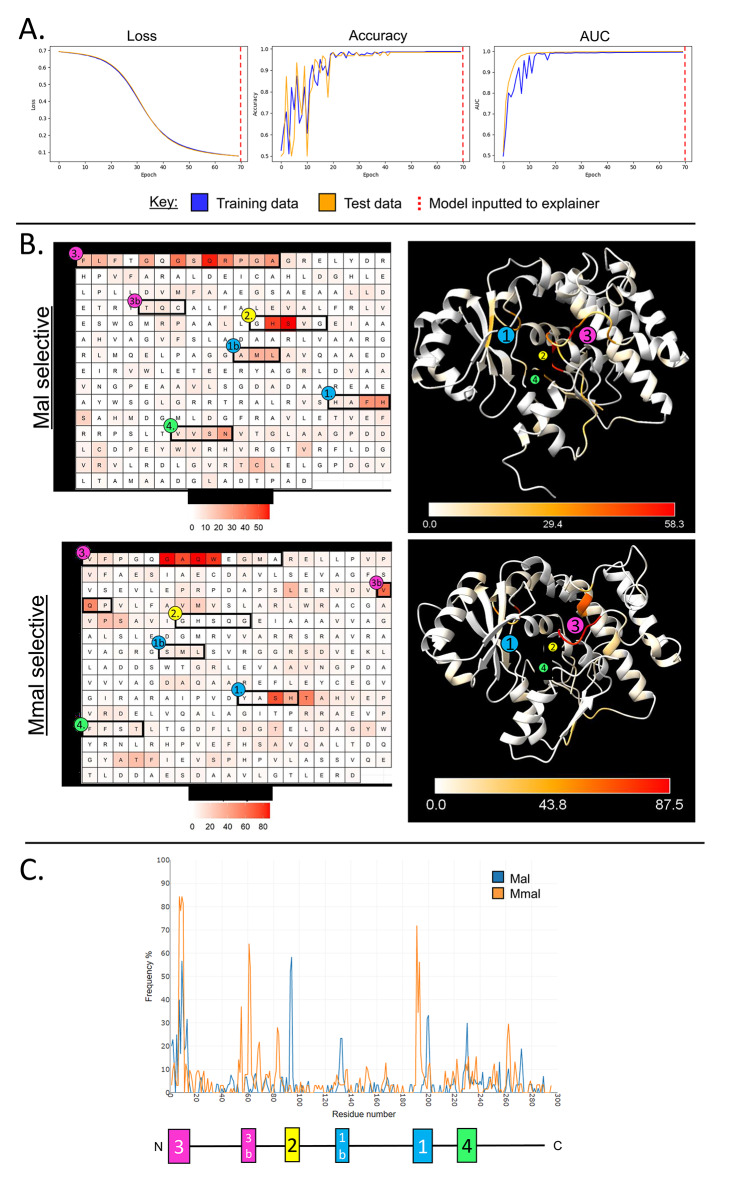
**(A)** AT domain binary classifier training and test data loss, accuracy and AUC over a 70 epoch training cycle. **(B)** Raster pattern, heat maps of Malonyl-CoA selective (*Streptomyces venezuelae*, PikAI AT3^56^, MIBiG: BGC0000094) and Methyl-malonyl-CoA selective (*Saccharopolyspora erythraea*, EryAI AT2^57^, MIBiG: BGC0000055) sequences (left) and their corresponding AlphaFold structures (right). The heat scoring in this panel displays the frequency a residue position was detected by the explainer in the model’s test partition (see methods section). Sequences were chosen arbitrarily as class representatives. Overlaid boxes correspond to highly predictive regions within the active site pocket and motifs experimentally validated as influential in substrate specificity prediction (1 and 2)^51–54^ and surrounding the active site pocket (3 and 4). We label 1b and 3b as hot regions contacting 1 and 3, which may be detected through the convolutions. **(C)** Frequency scores corresponding to residues in the panel B.

To validate the utility of our machine learning pipeline, we ran a pilot study using AT domain substrate specificity as an equivalent task. *Cis*-AT, mPKS AT domains are predominantly substrate specific for either Mmal or Mal^50^, which we also found in our dataset (see supplementary Figure S2). Motifs influential to this specificity have been experimentally demonstrated as YASH and GHSQG for Mmal, and HAFH and GHS[I/V]G for Mal^51–55^. Further mutations to a Mmal-specific RVDVV motif immediately preceding a conserved glutamine facing the active site (Q66 and Q61 in Fig. 1B, box 3b) have also been shown to be weakly substrate selective^51,55^.

As an essential domain to *cis*-AT mPKS modules, AT domains could be mined from the same BGCs as the KSs at a similar scale, and so would provide a machine learning task where we know that features of the structure correlate to substrate specificity. Predicting AT substrate specificity was therefore anticipated to be a task of a similar nature to predicting KS specificity and one where we could cross validate the model’s predictions – a method that can detect and explain AT specificity is likely to be applicable to KS proofreading, and we can judge this applicability through the detection of known motifs.

We trained a binary classifier GNN on 614 unique AT domain structures using an 80:20 training and test data partition split. Performance metrics of loss, accuracy and area under receiver-operator curve (AUC) for training and validation data, shown in Fig. 1A, are highly correlated, which indicates that the model has learnt meaningful information about the relationship between AT structure and substrate specificity. The model concluded with an accuracy score of 98% and an AUC score of 98%, indicative of excellent predictive performance. Following a 70-epoch training period, the test data partition was analysed using a model explainer to identify residues significant to the model’s predictions (Fig. 1B–C).

The AT binary classification model presented here can detect whether an AT structure is specific for Mal or Mmal with near perfect accuracy. As seen in the sequence heat maps (Fig. 1B), the model was able to detect the HAFH/YASH and GHS[I/V]G/GHSQG motifs, and the conserved glutamine (box 3b) and RVDVV motif^51^, demonstrating that our method can be used to correctly identify residues influential in determining substrate specificity. The model detected a further two regions, 3 and 4, that form the active site pocket. Previous mutagenesis studies attempting to alter AT substrate specificity using motifs in regions 1 and 2 found that specificity did not change by 100%^51, 53, 54^, and so there is scope for regions 3 and 4 to be influential in substrate specificity as well. Notably, the heat maps highlight region 3 as containing the most frequently detected substrate-specific residues.

### Counterfactual AT structures – substrate specificity motif swaps

**Fig. 2:**
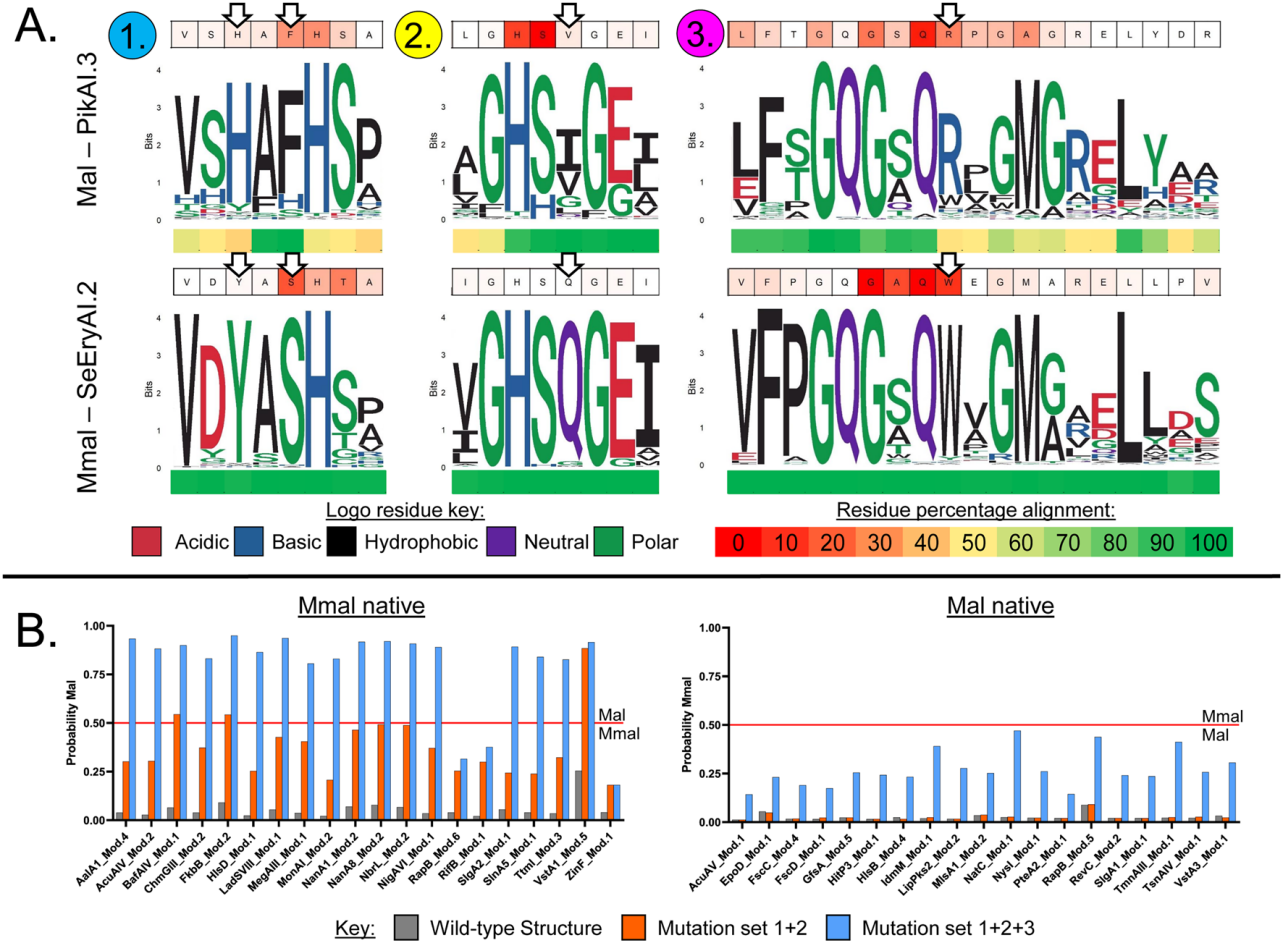
**(A)** 2.5 Å pairwise alignment logo diagrams of AT regions 1–3 and corresponding heat map scoring per Fig. 1B. Arrows indicate residues swapped to produce counterfactual structures. Colour bars under residues correspond to the percentage of residues aligning to the subject structure. **(B)** Comparison of prediction scores for wild-type and motif-swapped (modified) AT AlphaFold structures. The y-axes in both graphs correspond to the substrate expected for the modified structure, not the wild-type substrate. The red lines at 0.5 indicate the decision thresholds for the model to make a prediction of the modified substrate.

To interrogate the AT domain GNN further, we generated a partition of 40 sequences where the YASH/HAFH and GHS[I/V]G/GHSQG motifs were swapped to the opposite motif (20 from Mal to Mmal, and 20 from Mmal to Mal). AlphaFold structures were then produced from the altered sequences. This type of motif exchange has been demonstrated to change AT substrate specificity between Mmal and Mal^52–54^, and so we would expect to see a difference in the prediction scores from the GNN, when comparing against the predicted structure of the unmodified sequence (Fig. 2). A second set included these mutations, plus a W/R mutation, to explore the influence of region 3.

Results contained in Fig. 2B indicate that a W➔R or R➔W mutation in region 3 has a greater effect on substrate prediction score than those of the experimentally validated motifs in regions 1 and 2. We do not have experimental evidence to explain the role of W/R in substrate specificity and the significance of this position highlights a limitation of the method: residues that correlate with substrate specificity may not cause specificity and may be a product of a confounding variable, such as phylogeny. Figure 2B also shows that mutations to regions 1 and 2 only changed the prediction scores in the Mmal➔Mal structures. Considering the heat map scores for regions 1 and 2, hypothetically, a model would not have to learn that both GHSQG predicts Mmal and GHS[I/V]G predicts Mal; the same classifications could be achieved with GHS[I/V]G predicts Mal, not-GHS[I/V]G predicts no increase in Mal probability (whilst relying on another motif to predict Mmal). Together, this highlights that models can learn class-predictive patterns in an asymmetric manner and an awareness of this is important to interpreting the heat maps for purposes of mutagenesis.

Also of note is that conserved residues can present as hot when proximal in 3D space to class specific residues (e.g. region 3’s Q next to the W/R in Fig. 2A). Convolutions in a GCN layer pass information about a subject node to its edge-joined neighbours, which allows for descriptions of neighbourhoods and interactions to be learnt by the network. We therefore suggest that it is likely the significance of these conserved residues is that they contextualise the variable neighbour. Removing these contextualising residues would likely modify the detection of the variable residue, which would lead to a false change in classification of the structure.

### Ketosynthases substrate specificity models

#### β-carbon reduction state binary classifiers

**Fig. 3:**
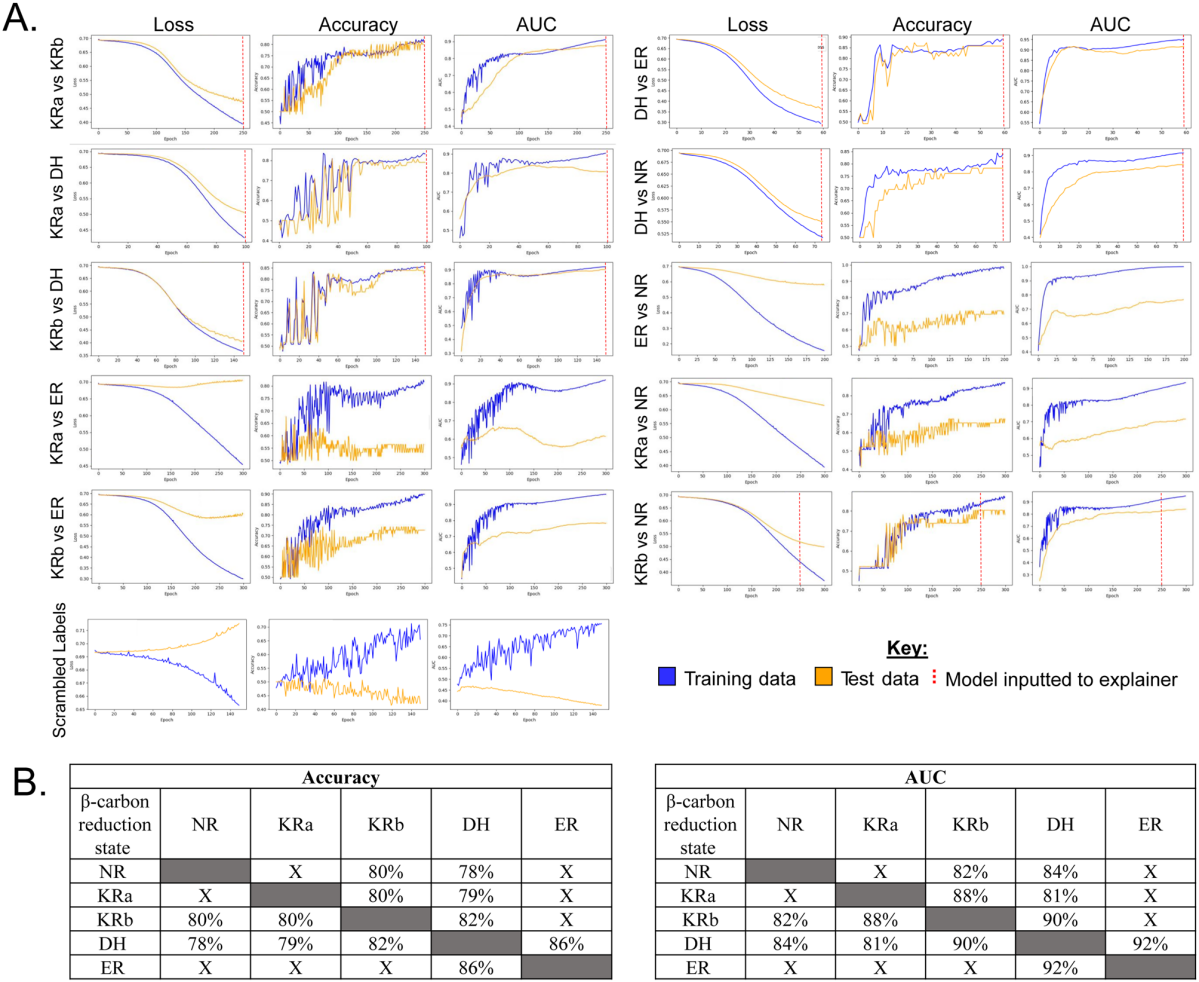
**A**: Training performance metrics of loss, accuracy and AUC for KS β-carbon binary classification models. Variable axes have been used here to better illustrate the relationship between test and training data performance. **B**: Test data partition accuracy and AUC scores for β-carbon reduction state, corresponding to stopping points in the graphs above. An “X” result indicates that the model was rejected for poor performance on the test partition.

We ran ten binary classification models, examining β-carbon states in a pairwise manner (Fig. 3). Substrate labels were based on the β-carbon moiety of the native polyketide substrate: Ketone (NR-type, 122 structures), l-alcohol (KRa-type, 161 structures), d-alcohol (KRb-type, 159 structures), alkene (DH-type, 387 structures; combining 8 *cis* and 379 *trans* alkenes) and methylene (ER-type, 166 structures). Training and test data for each model were balanced according to the minority class for each pairwise comparison.

A final classifier was run on KRb-type and DH-type KSs, with labels randomly assigned (scrambled), to control for the neural network’s capacity to overfit KS structural data (i.e. learn idiosyncratic details of the training data that do not generalise to the underlying classes). Overfitting is assessed using held-out test data, with overfitting inferred from the loss of performance on the test data relative to the training data in Fig. 3A.

**Fig. 4:**
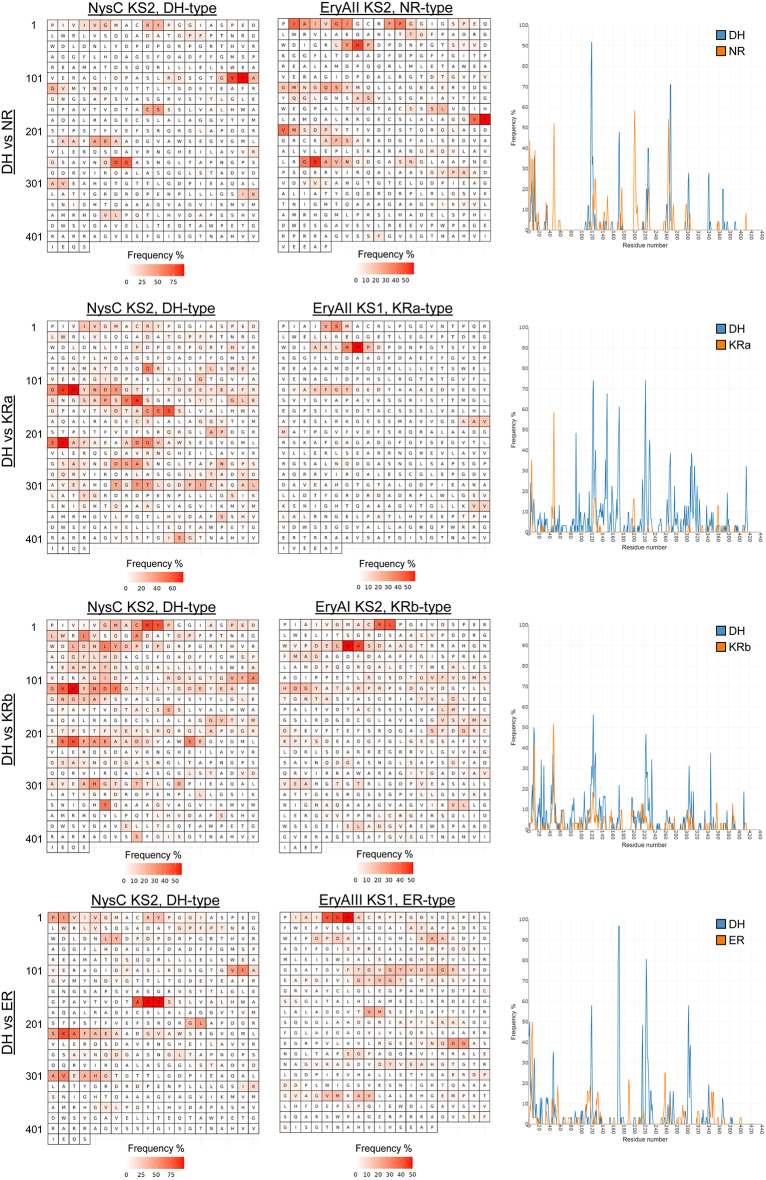
Aligned, monomeric explainer heat maps (left) and corresponding frequency scores (right) for models trained with DH-type KSs. Heat scoring displays the frequency a residue position was detected by the explainer in the model’s test partition. The sequence maps on the left are a raster pattern from left to right, top to bottom of the primary sequence of a KS of a β-reduction class. Sequences are sourced from *Saccharopolyspora erythraea* (erythromycin, MIBiG: BGC0000055)^57^ and *Streptomyces noursei* (nystatin, MIBiG: BGC0001709)^58^. Colour scales used in heat maps are not comparable between maps.

**Fig. 5:**
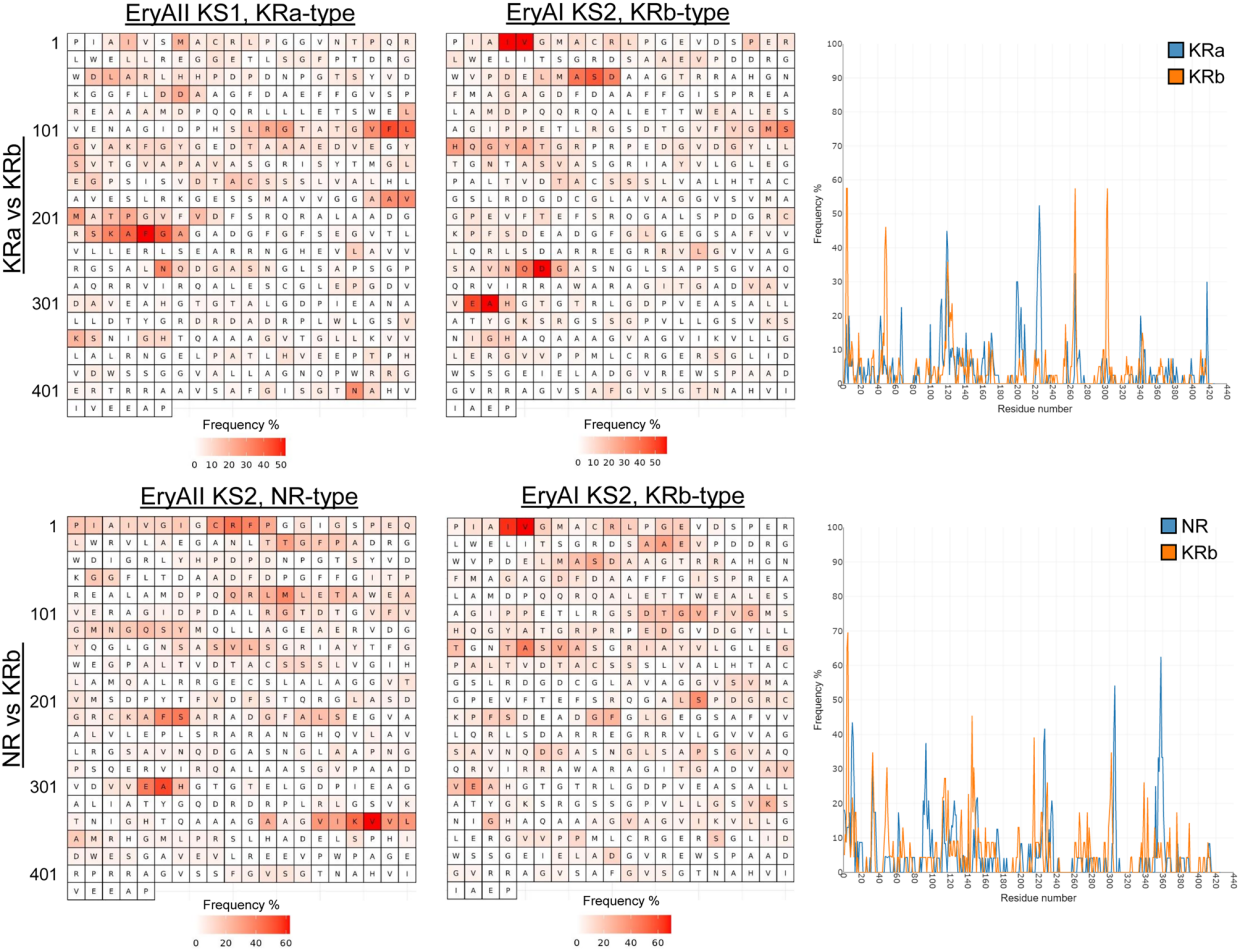
Aligned, monomeric explainer heat maps (left) and corresponding frequency scores (right) produced from the KRa vs. KRb and NR vs. KRb model. Heat scoring displays the frequency a residue position was detected by the explainer in the model’s test partition. Sequences are sourced from *Saccharopolyspora erythraea* (erythromycin, MIBiG: BGC0000055)^57^. Colour scales used in heat maps are not comparable between maps.

The binary classifiers presented in Fig. 3 most consistently distinguished KS structures expecting an alkene at the β-position (DH-type). KRa and KRb-type KSs were distinct from each other, as were KRb-type KSs from NR-type KSs. All models outperformed the scramble control, though pronounced overfitting was observed in the KRa vs. ER, KRb vs. ER, ER vs. NR, KRa vs. NR models – these models were therefore not considered further. The remaining six models were taken forward and used to produce the heat maps in Figs. 4 and 5.

Compared to the AT domain pilot, KS classifiers returned lower AUC scores, indicating weaker predictive performance and less reliable separation of substrate classes. Divergence of test and training metrics is also more pronounced in the KS classifiers, indicating a degree of overfitting – this likely presents as noise in the heat maps in Figs. 4 and 5. This difference likely reflects both methodological and biological factors. In particular, the training labels may not fully capture the relationship between KS structure and substrate specificity. While AT domains typically exhibit well-defined and discrete substrate preferences, KS domains can tolerate a range of substrates, suggesting that their specificity is better represented as a continuum rather than as mutually exclusive classes. Previous mPKS modification experiments that resulted in reduced turnover^16–18^, rather than stalling, indicate that KS proofreading can operate on a spectrum, and promiscuity in KS substrate specificity has been identified in the Rapamycin and Mycolactone mPKSs^18,29^. In the present study, KSs were represented using a single native substrate label due to limited data on substrate tolerance, meaning that promiscuous and highly specific KSs were treated equivalently. While this assumption is supported to some extent by β-carbon-associated sequence motifs^29^ and the trends observed in Fig. 3B, it likely limits the ability of the model to capture functional nuance in vivo.

In the supplementary material, we analyse the relationship between phylogeny and the polyketide substrate’s β-carbon moiety. Our results recapitulate previous studies showing that KSs group by taxonomy and form subclusters by substrate type^15,23,28^. Supplementary Figure S10D shows that for the majority of KSs in all type pairings tested, individual BGCs will contribute structures for both types and so learning taxonomy-specific features would be counterproductive to model performance in most instances.

### Interpretation of DH vs. ER model and its application to altering substrate specificity in vivo

To explore if the GNNs can be used to predict mutations that modify substrate specificity in favour of a non-native β-carbon moiety, we ran a proof-of-concept experiment on the DEBS mPKS from the *S. erythraea* erythromycin pathway^57^, expressed in *E. coli* without tailoring reactions. This pathway is considered a model mPKS pathway and one where KS mutagenesis studies have been conducted^23,24^. Here we knocked out the ER domain in module 4 to change the substrate received by the downstream KS (EryAIII KS1) from a β-methylene to a β-alkene. A pathway schematic is included in supplementary Figure S11. This mutation has been described by Donadio et al.^46^ as reducing productivity in the pathway by approximately 5-fold, which we suspected may be a product of proofreading in EryAIII KS1.

**Fig. 6:**
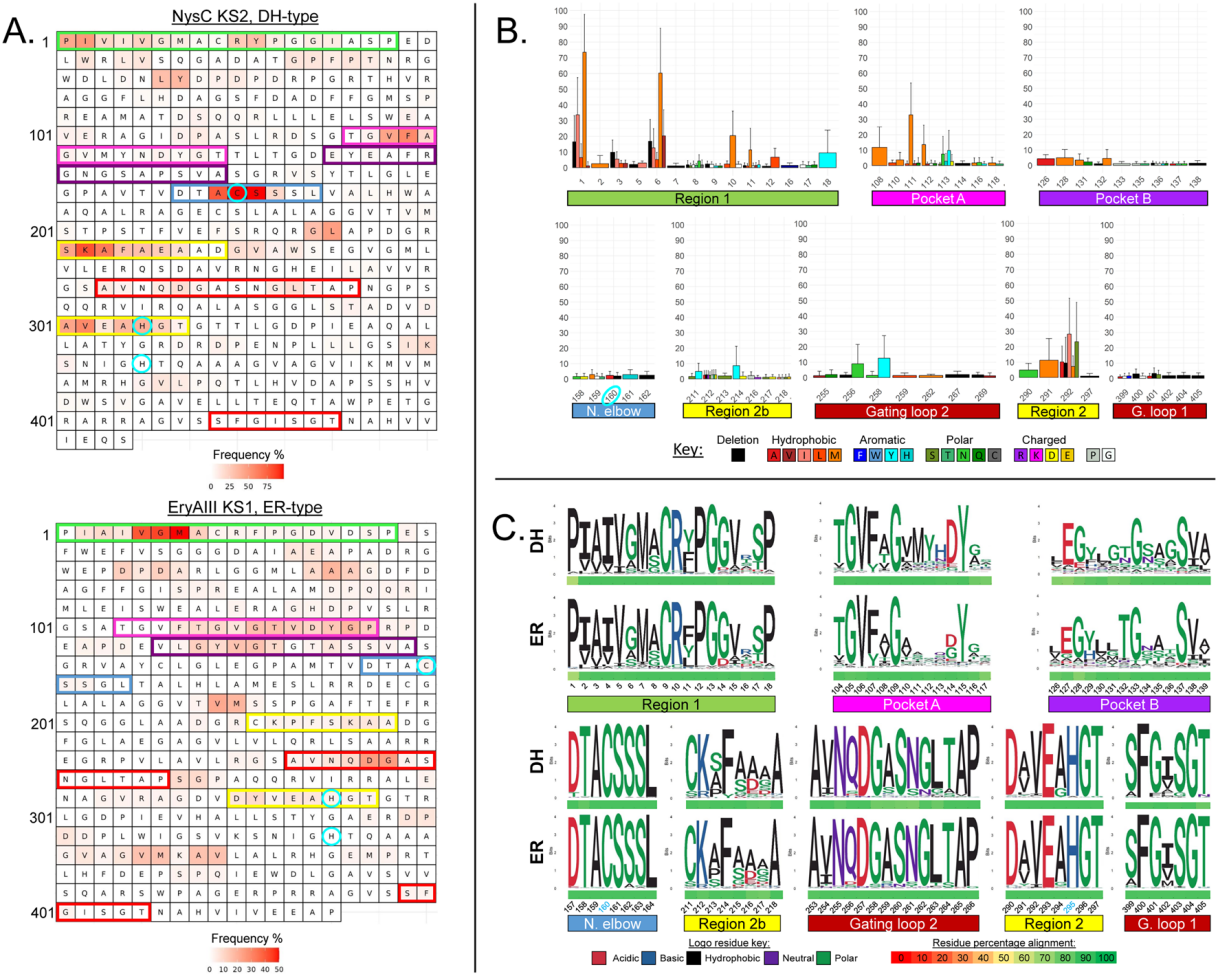
**(A)** DH vs. ER model heat maps with overlay of regions explored for mutagenesis. Overlay colouring corresponds to the bars beneath graphs in panels B and C. **(B)** Single residue changes for mutant structures, filtered for predictions greater than 1% to ER-type. Averages are calculated from the difference between the models score for the wild-type EryAIII KS1 structure and the mutants. Bars display standard deviation. **(C)** 2.5Å proximity pairwise logo diagrams. Alignments for DH and ER were conducted against NysC KS2 and AceP2 KS3 (*Couchioplanes caeruleus*^60^, MIBiG: BGC0001491.1) – this was used because EryAIII KS1 aligns poorly (possibly due to being 8 amino acids short of the average length of a KS domain in the training data). Residue numbering under logos and bar graphs correspond to the position in the EryAIII KS1 sequence in panel A.

To identify mutation candidates that could rescue this substrate change, we opted to focus on residues/structural regions that have known function and test simulated mutations via AlphaFold structures in the DH vs. ER model. Regions explored have been displayed in Fig. 6 and include the post-cysteine substrate pocket (including the dimer interface loop^29^), the gating loops^11,25,59^, and a selection of hot residues proximal to the active site cysteine and EAHGTG histidine^9^. We also include region 1, which appeared in all heat maps produced for KS classifiers in this study, though we do not have an explanation for why it is being detected. The Figs. 4 and 5 heat maps have been annotated in the supplement for comparison of these regions across models (Figures S12–13), along with per-class structure-based logo diagrams (Figure S14–18). Mutations tested on the model were based on the set of amino acid-types detected in a 2.5 Å proximity alignment at the subject position, across the DH class (N.B. this includes infrequently detected residues that are poorly visible in the Fig. 6C logos). Mutant AlphaFold structures were analysed by six independently trained DH vs. ER classifiers using randomised training data allocation to create the averages displayed in Fig. 6B. Multiple models were used was to minimise noise and capture variations created by the random data partitioning of training data.

The 2.5Å proximity logo diagrams in Fig. 6C confirm some residue change predictions in 6B as explicable, most notably G111M in pocket A, which falls within a previously described VMYH motif^29^, V292S/I in region 2, and F214Y in region 2b, which is located directly behind the histidine in region 2. Others, most notably residues P1M and G6M, are not well explained by the logos. Also of note is a + 2% C160A prediction on the active cysteine, which would result in a catalytically inert KS, highlighting a need to sense-check predictions. Given the essential and invariant nature of C160, we suggest this residue might be providing context to neighbours (for example, G111 is within 2 k-hops of C160).

**Fig. 7:**
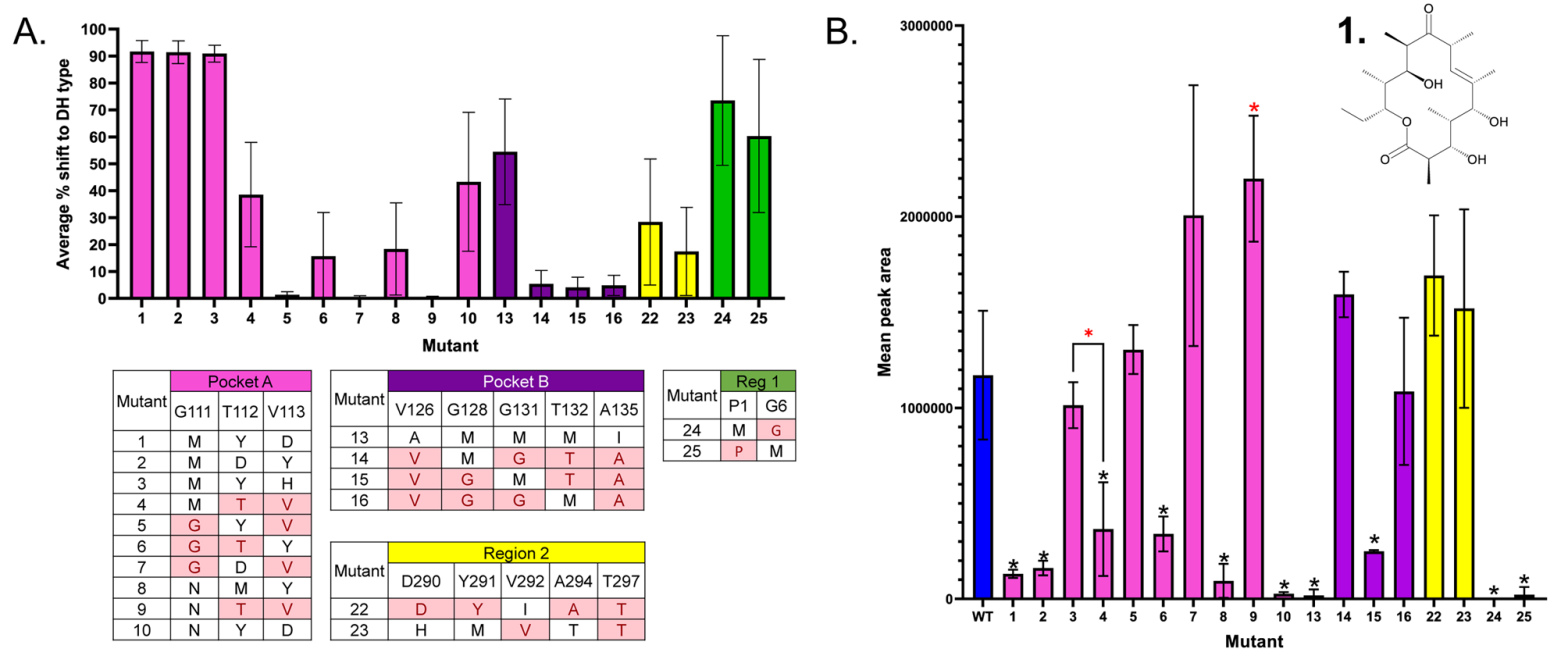
**(A)** EryAIII KS1 mutations tested in vivo and corresponding predicted average shift to DH-type for AlphaFold simulated mutations. Bars indicate standard deviation **(B)** Average peak area for compound **1** hydrogen adduct mass range +/- 2ppm, at retention time 590–600 s. Bars indicate standard deviation. Asterisks indicate t-test p values below 0.05 when compared to WT. A second comparison between mutants 3 and 4 is also displayed. Corresponding extracted ion count graphs for compound **1** are shown in supplementary Figures S19 and S20.

We next expanded our search to explore combinatorial effects on predictions. Figure 7 displays a selection of single and combinatorial mutations implemented in vivo and their corresponding mass spectrometry results for the production of the expected ER knockout DEBS product, compound **1 (**6,7-anhydroerythronolide B ^46^). Mutants 1–4 explore combinations including the high scoring G111M mutation. Here, mutant 3 contains the consensus MYH. Compared to the wild type (WT) KS, this set of mutations resulted in 13% less compound **1**. However, the MYH mutation did produce 2.7x more compound **1** than mutant 4, which features G111M only, indicating contextual sensitivity for a methionine at this position. Other high scoring combinations MYD and MDY (mutants 1 and 2) performed much worse against the WT. Mutations 8–10 considered G111N associated mutations, which was detectable when provided context of T112 and V113 mutations. An asparagine at position 111 is weakly detected in Fig. 6C; however, this type of mutation would be considered consensus for a KRb type^29^. It is therefore interesting that mutant 9 was the best performing mutant tested, producing nearly 2x more compound **1** than the WT. We tested pocket B with methionines at positions 128, 131 and 132 in mutants 13–16. Methionines here are not well supported by the logos in Fig. 7C, detectable 3, 19 and 3 times at positions 128, 131 and 132 respectively in the 387 DH-type structures. Residues linking pocket A to pocket B were observed to vary in length between KS sequences in the training data, though we did not find any difference in the average length of KS sequences between classes. Collectively, mutants 13–16 indicate that a methionine in pocket B was learned as more DH-like, which mutant 14 (1.4x more compound **1** than WT) suggests could be valid, though overemphasising this signal can trick the model, shown by mutant 13. This reiterates an important property of these models – they were trained to predict whether a natural structure is more likely to be a DH or ER-type, and not to detect whether a mutation is viable. Mutant 22 produced 1.45x more compound **1** than the WT, which suggests that modification of the structure behind the histidine in the EAHGTG motif can be beneficial. Region 1 remains confounding.

### Extension unit and α-carbon binary classification

Considering substrate specificity beyond the β-carbon, KS domains variably encounter α-methylation on the polyketide substrate, depending on the upstream AT domain’s substrate selection (or in rarer cases, a methylase domain^9^). Additionally, there is the extension unit substrate supplied to the condensation reaction by the relative C-terminal AT domain, which structurally adjoins the subject KS. Using the KS-last module border definition^15,22^, we would expect that if substrate specificity can be detected, it would be on the polyketide and not the extension unit.

**Fig. 8:**
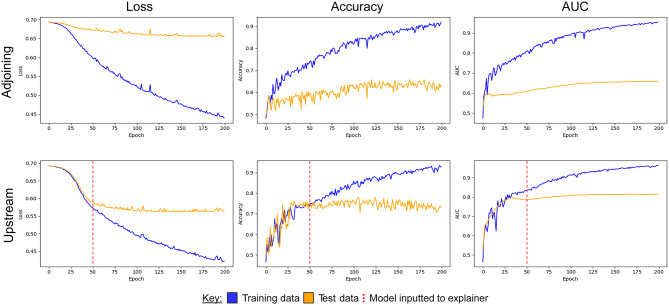
Training metrics of Loss, Accuracy and AUC for binary classifiers trained on ketosynthase dimers, using either the upstream or structurally adjoining AT domain’s choice of Mmal or Mal as labels.

We trained two binary classifiers on KS structures to predict AT substrate choice of Mal or Mmal as classes (Fig. 8). The first model assigned classes based on the substrate specificity of the relative C-terminal, extension unit supplying AT domain. This model was trained on 720 KS structures. A test partition of 180 non-redundant structures produced an AUC of 64% and 63% accuracy after 100 epochs. Given that a random classifier should return AUC and accuracy scores around 50%, this model indicates poor predictive performance and the model was not taken forward.

**Fig. 9:**
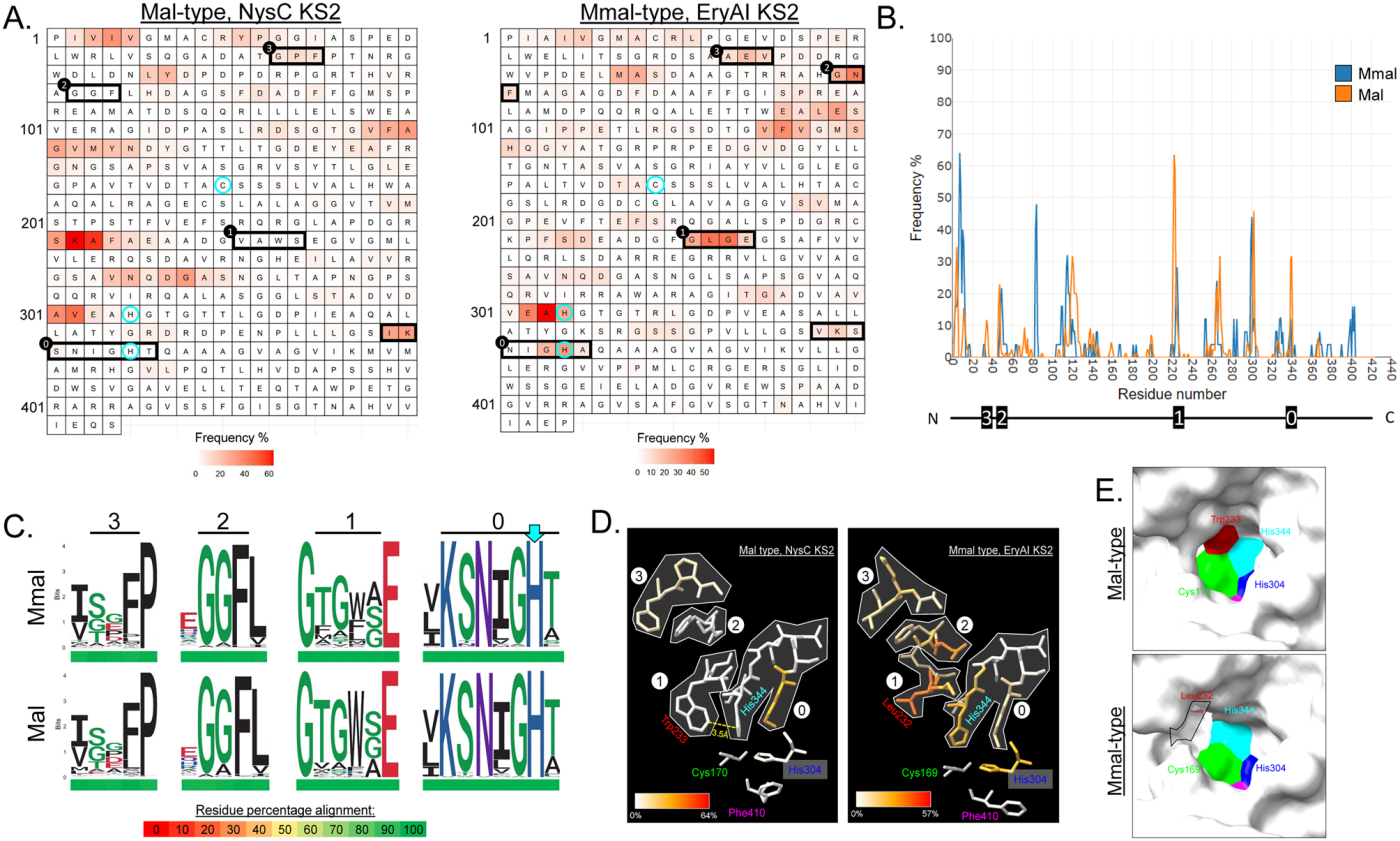
Analysis of upstream extension unit (α-methylation) model. **(A)** Aligned, monomeric explainer heat maps. Heat scoring displays the frequency a residue position was detected by the explainer in the model’s test partition. Sequences are sourced from *Streptomyces noursei* (nystatin, MIBiG: BGC0001709)^58^ and *Saccharopolyspora erythraea* (erythromycin, MIBiG: BGC0000055)^57^. The region labelling applied to the heat maps highlights a series of residues detected at approximately 1, 2 and 3 k-hops from the KSNIGHT motif (0). **(B)** Frequency scores corresponding to panel A. **C**: 2.5Å proximity pairwise logo diagrams using NysC KS2 and EryAI KS2 as the alignment subject. **D**: Atomic structure view of k-hops 1–3 from KSNIGHT, 0, as well as active site catalytic residues. Colour coding uses heat map scoring. **E**: Surface view of active site tunnel from ACP docking face. Phe208 has been hidden in Mmal-type.

The second model assigned classes based on the extension unit used in the upstream condensation reaction, relative to the subject KS. In effect, using Mmal/Mal labels here describes α-carbon methylation on the polyketide substrate. This model was trained on 842 KS structures that are not preceded by a methylase domain. A test partition of 168 structures produced an AUC of 79% and 75% accuracy after 50 epochs, where the model was stopped and analysed using the explainer (Fig. 9).

The training data used in both models share a substantial overlap in label assignment; 604 KSs in both sets have the same upstream substrate as the adjoining substrate (405 Mal, 199 Mmal). For the adjoining model, 44% of the minority category (Mmal) will share the same labels as in the upstream model. Assuming that the 75% accuracy score from the upstream model applies here, we would expect that 33% (75% of 44%) of the minority category can be predicted using the upstream substrate. Applying a random assignment to the remaining 56%, the model would have an accuracy score of 61% on Mmal predictions. Depending on the random sampling for training data, this would be slightly higher for Mal on average (see supplementary Figure S5). This could explain the performance observed in the adjoining model. However, this applies in both directions, and there may be some contamination in the upstream model where predictions are an inference of the downstream substrate.

Based on the test data performance from these models, we conclude that the upstream AT domain’s substrate choice is moderately predictable from KS structure.

The upstream extension unit model’s explainer results, shown in Fig. 9A, identify hot residues in the regions explored in Fig. 6. Expanding our search, the heat maps also identify a region behind the KSNIGHT motif’s active histidine, relative to the substrate pocket. Analysis of the variance in these residues highlights a tryptophan k-hop 1 from the histidine (Fig. 9C) that is more prevalent in the Mal-type (W233). This tryptophan is predicted by AlphaFold as proximal to the active histidine of KSNIGHT (3.5 Å between H344’s imidazole and W233’s indole ring in NysC KS2), as well as contributing to the substrate pocket’s morphology.

## Discussion

As a variable component in mPKS assembly lines, AT domain substrate specificity can be used to introduce chemical diversity to polyketides. These domains have been targeted for mutation in previous studies to introduce alternate or exotic (e.g. fluorinated^61^) extension units^52,54^. We suggest that the residues identified in Fig. 1 would make for interesting targets in similar future experiments.

Whilst the AT domain model can accurately discriminate between Mal and Mmal-type structures (Fig. 1), AT domains may not be the only determinant of extension unit fidelity within a given pathway, with KR^62^ and KS domains (Fig. 9 and Hirsch et al. 2021^29^) implicated in substrate specificity towards the extension unit choice post-condensation. Rare instances of promiscuity have also been identified within AT domains^63^. The KS and AT models we present in this study consider domains in isolation, and so translation to a biological context may be confounded by other domains in the mPKS.

Our KS binary classifiers find that six out of ten pairwise combinations of polyketide substrate β-carbon moieties can be predicted from KS structures with reasonable accuracy. Of these, classifiers containing the DH and KRb-types performed best – this coincides with these classes having more distinct fingerprint motifs in pocket A, described in the sequence-based logo diagrams in Hirsch et al. 2021^29^ and recapitulated in the 2.5 Å logos in supplementary Figures S14–18. However, these motifs do not fully explain the hot spots in Figs. 4 and 5. Polyketide substrate α-carbon methylation is also moderately predictable from the KS structure, whereas prediction of the extension unit used in the subject KS condensation reaction was unsuccessful (Fig. 8). This provides evidence in favour of the KS-last model of mPKS module borders^15,22^.

Our model-guided mutations experiment demonstrates that the GNNs presented in this study can act as a co-pilot for mitigating KS proofreading by providing target suggestions for mutagenesis. The results from the in vivo mutagenesis experiment indicate that these models struggle to precisely place where a mutation should go, as observed from the pocket A and B mutants in Fig. 7, and this can be exacerbated by cumulative mutations. Whilst these models can make useful predictions, they still require biochemical disambiguation, and the KS results should be interpreted as exploratory and hypothesis generating, rather than definitive.

The G111M set of mutations (mutants 1–4) represented both the highest scoring model predictions for the DH-type and the most distinct against the ER-type in the logo diagrams. This highlights that both the GNN and sequence-based approaches do not always translate to the desired biological outcome. However, comparing the methods, GNNs offer a contextually sensitive approach that operates on 3D structure and the relationship between residues beyond primary sequence – therefore this method offers insights closer to the protein’s form-function relationship than a purely sequence-based approach. Where we find the GNN approach to be particularly advantageous is in the detection of residues that are less obvious from sequence alignment approaches, as shown with mutants 14 and 22 (Fig. 7). Our mutagenesis pilot explored regionally confined sets of mutations and the results indicate that a second round of mutations combining regions might be beneficial (e.g. V292I + G111N), were we to seek further improvements in substrate specificity.

For those interested in using these models in their own research, we have included a simplified Python script and instructions for using the classifiers to make predictions on substrate specificity on our GitHub page. For more advanced users, AlphaFold structures used as training data and scripts for training classifiers are also available on GitHub.

Our method of model disambiguation focussed on regions and residues that could largely be explained by sequence variation, though this does not explain all the regions in the heat maps (Fig. 6). Some of these unexplained areas may reflect the limited scope of our mutagenesis pilot: we examined the DH vs. ER model in only one direction of change, and within the context of a natively β-alkene–tolerant KS, making it unlikely that we captured the full extent of what the model learnt about the substrate–structure relationship. Likewise, our choice of ER catalytic knockout over domain deletion will not have captured nuances that may arise from module-level structural differences. Model interpretation is a significant challenge in the wider field of machine learning and whilst the model explainer we used in this study^41^ highlights residues that are important, it does not capture the reasoning. Considering the heat map results further, the GNNs employed here may be able to learn more advanced rules than were explored in this study and this may underpin why sequence-conserved regions are detected. For example, a GNN could identify two proximal conserved motifs if their relative positions vary between classes, which would not present in the primary sequence of the conserved motifs. If this relative positioning is the product of an insertion or deletion, or change to secondary structure orientation, the cause may be less pronounced than the effect in the network graph’s structure, and so the effect rather than the cause is chosen for classification. Such scenarios highlight the value of a GNN for detecting subtle differences over sequence-based methods, but software for detecting and visualising this information in an interpretable format is a limitation – we captured some of this information through the 2.5 Å alignment method though a more comprehensive format in 3D is needed.

Comparing the KS heat maps across models (supplementary Figures S12–13), we observe recurring patterns of hot residues, notably in the gating loops and substrate pocket. We find the detection of the gating loops to be particularly interesting, given their role in substrate selection in fatty acid synthase (FAS) KSs^59^. The 2.5 Å alignment logo diagrams in Fig. 6C indicate greater variance in the DH-type, notably in residues 263–265 (loop 2) that are proximal to residue 402 (loop 1), which takes the place of the gating phenylalanine in FAS KSs^59^. A methionine at position 402 presents as more ER-like from the logos in Fig. 6C. A mutagenesis study has implicated residue 264 (S315A in Robbins et al. 2016^25^) in translocation and chain elongation, and so we suggest that these loops would be a good choice for an expanding the mutagenesis study presented here.

We were unable to produce models that reliably distinguish ER-type KSs from NR and both KR-types, and KRa-types from NR-types (Fig. 4) – whether this was due to insufficient training data, the structures fundamentally being too similar/noisy to distinguish using our GNN method, or that there is an underlying biological or chemical explanation, we do not know. Our suggestion is that these four classifiers would be worth retraining at a future date where more non-redundant, annotated KS structures are identified.

In this study, we demonstrated that structure-function relationships can be uncovered in highly related protein structures using GNNs, and these can be used to generate actionable targets for mutagenesis. We therefore suggest that the method and approach described here may find broader utility in protein engineering, particularly on tasks where discrimination between highly similar structures is required. A limitation of our method is that we were exploiting the correlation between sequence and function to extract substrate-altering mutation predictions, rather than training the models to learn determinants of substrate specificity proportionately. This approach therefore struggles in the ranking of mutations. Furthermore, the use of mutually exclusive labels presents a barrier to learning promiscuous mutations^23^. Future expansions of this work might therefore benefit from using an alternative labelling format that describes specificity as a continuum rather than discrete categories – such continuous labels would need to be derived biochemically, through either experimentation or simulation.

## Supplementary Information

Below is the link to the electronic supplementary material.

Supplementary Material 1

## Data Availability

Code, AlphaFold structures and substrate labels produced for this study have been made open access on GitHub: https://github.com/mwalm/ketosynthases.
