# Supplementary material for: Graph neural networks can predict ketosynthase substrate specificity
Source: Sci Rep. 2026 May 9;16:21342. doi: 10.1038/s41598-026-47323-x (PMC13347036; doi:10.1038/s41598-026-47323-x)
Supplement: Supplementary file 1 — Supplementary Material 1 [file 41598_2026_47323_MOESM1_ESM.docx]

Supplement: Graph neural networks can predict ketosynthase substrate specificity

Table S1: 109 BGCs sourced from ClusterCAD^1^ for use in this study. Sequences and structures are available on the associated GitHub page: <https://github.com/mwalm/ketosynthases>

| **Domain type** | **Total** |
| --- | --- |
| Acyltransferases (AT) – total | 971 |
| - Mmal-AT | 359 |
| - Mal-AT | 529 |
| - Other-AT | 83 |
| Ketosynthases (KS) - total | 1138 |
| - NR-type | 122 |
| - KRa-type | 161 |
| - KRb-type | 159 |
| - DH-type | 387 |
| - ER-type | 166 |
| - Edge cases | 143 |

Table S2: Summary of total protein structures used in this study. Note that where a reductive state is being given for a KS, this is referring to the β-carbon on the incoming polyketide to the condensation reaction. The difference between the total numbers of AT and KS is a result of using an expanded range of BGC for the KS structures. KS reduction state is assigned based on the presence of both the associated reductive domains and inferred activity in the chemical product of the pathway. Edge cases include decarboxylative KS_Q_ and KSs immediately following a loading module.

Figure S3: Frequency of modules per mPKS pathway within dataset. Note: Only modules that contain a ketosynthase are counted. AT-ACP loading modules are not counted. Two pathways are NRPS hybrids that only contain a single PKS module (Nostopeptolide and Glidobactin). 1083


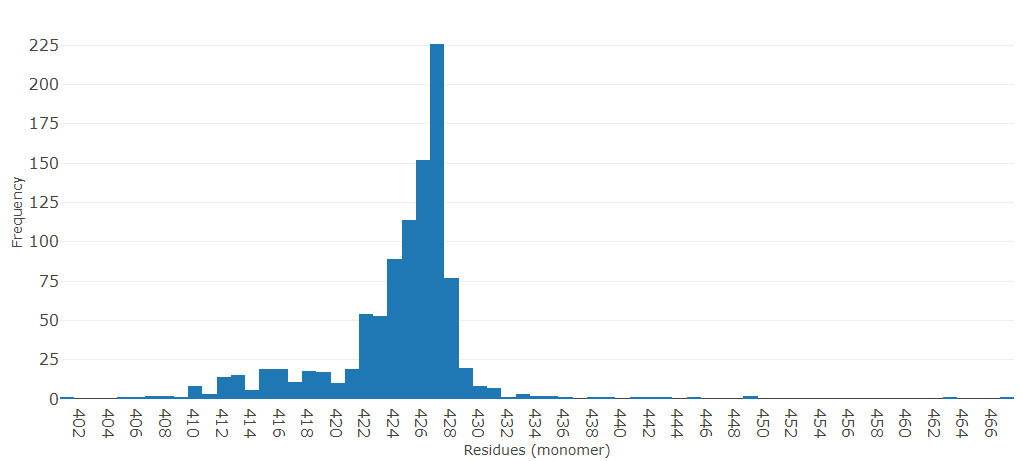


| **Average sequence length per class** | | | | | **Average total** |
| --- | --- | --- | --- | --- | --- |
| **NR** | **DH** | **ER** | **KRa** | **KRb** |  |
| 423 | 424 | 423 | 425 | 425 | 424 |

Figure S4: Frequency of residues per ketosynthase monomer in total dataset (top) and average sequence length divisible by β-carbon moiety (bottom).

Figure S5: Distribution of labels used in ketosynthase extension unit classifiers.


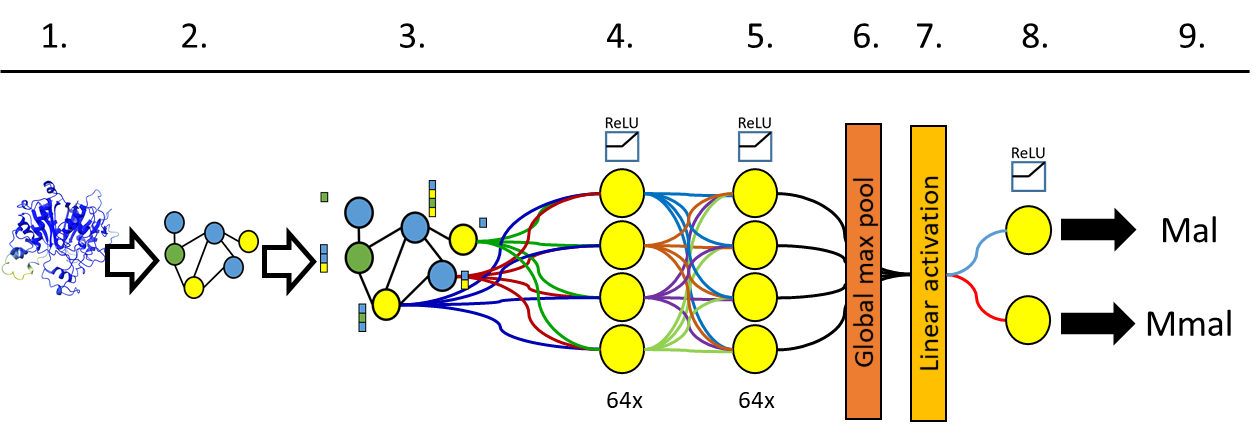


Figure S6: Process map and network architecture for AT domain binary classifier model. AlphaFold structures are converted into network graphs (1–2) and run through a two-layer GCN (3–5). Outputs are then pooled (6) and run through a linear activation (7–8) to give a prediction of Mmal or Mal.


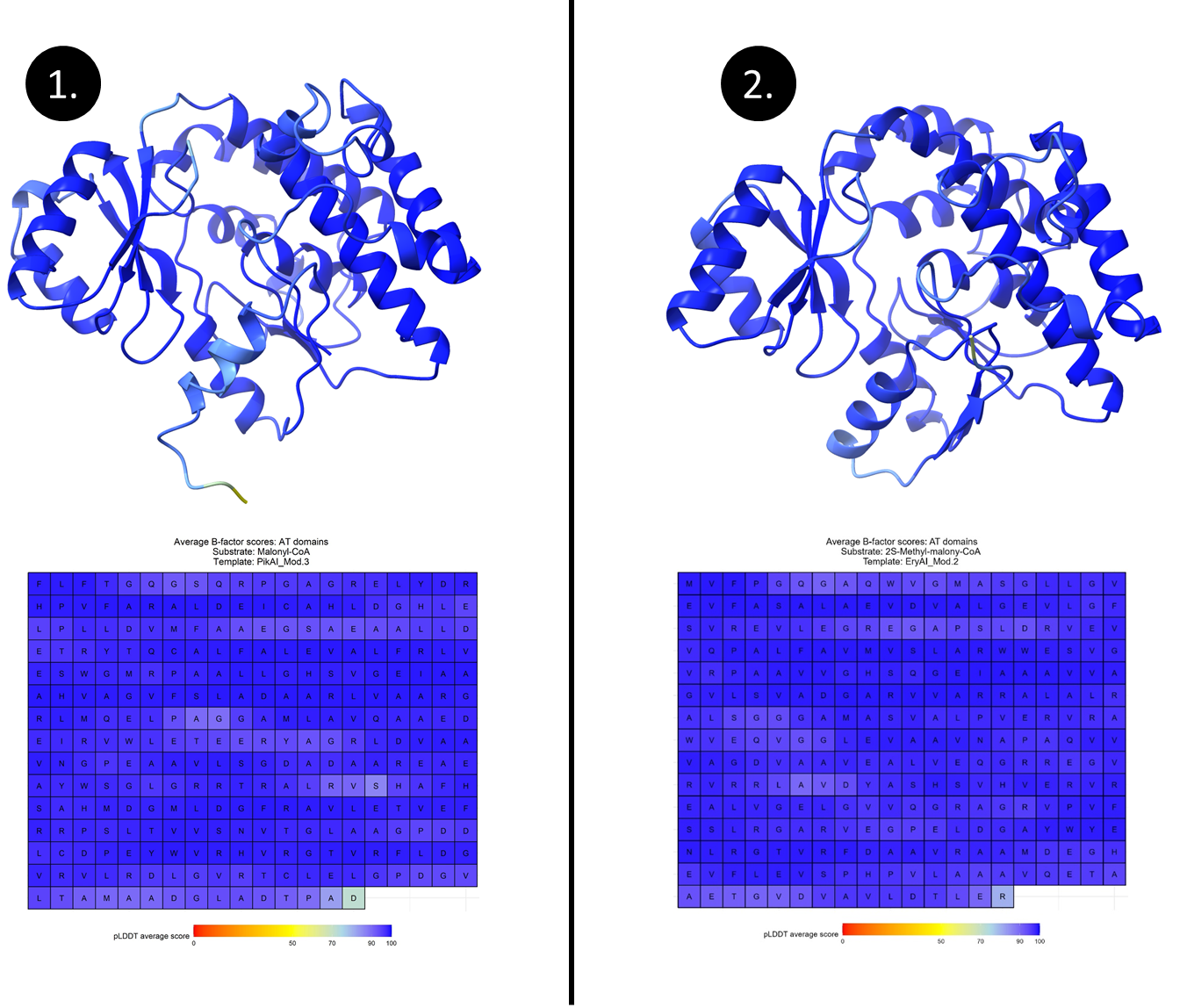


Figure S7: Total dataset average β-factor scores for **1.** Malonyl-CoA selective (n=529) and **2.** 2*S*-Methylmalonyl-CoA selective (n=359) AT domains. Alignments have been performed against PikAI.3 and AeEryAI.2 respectively. Top panels show the 3D structures, bottom panels display word search diagrams. Colouring in both diagrams follows AlphaFold’s pLDDT colour scheme^2^.


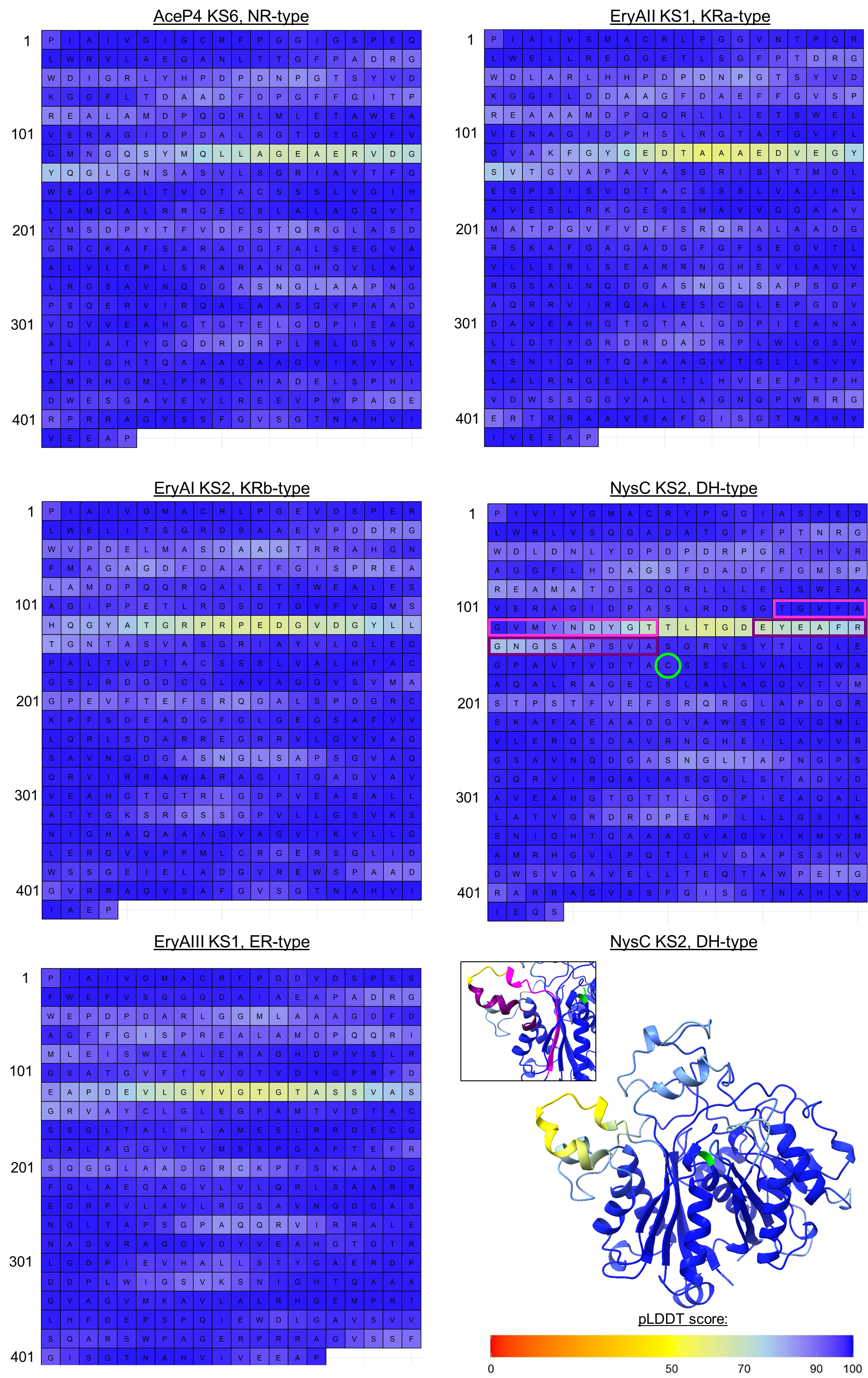


Figure S8: Average pLDDT scores for KS dimers, subdivided by incoming polyketide substrate β-carbon reduction state. KRa (n = 161), KRb (n = 159) and ER (n = 166) sequences are sourced from the *Saccharopolyspora erythraea* (erythromycin, MIBiG: BGC0000055)^3^, DH (n = 387) from and *Streptomyces noursei* (nystatin, MIBiG: BGC0001709)^4^, and NR (n = 122) from KS3 (*Couchioplanes caeruleus,* MIBiG: BGC0001491.1) ^5^. Averages generated by 2.5Å proximity method. **Bottom right:** NysC KS2 monomer (viewed from dimer interface) with average pLDDT scoring for DH-class. Cysteine in TACSSS domain has been highlighted in green. In the top left of the structure panel, pockets A (magenta) and B (purple) have been coloured to match the overlay in the DH-type pLDDT panel above. Colouring in both the heat maps and the structure diagram follows the same pLDDT colour scheme.

# Phylogenetic analysis of KS domains

Figures S9­–10 display phylogenetic trees (S9) and corresponding statistical analyses (S10) of the β-carbon KS-type pairings used to produce the binary classifiers.


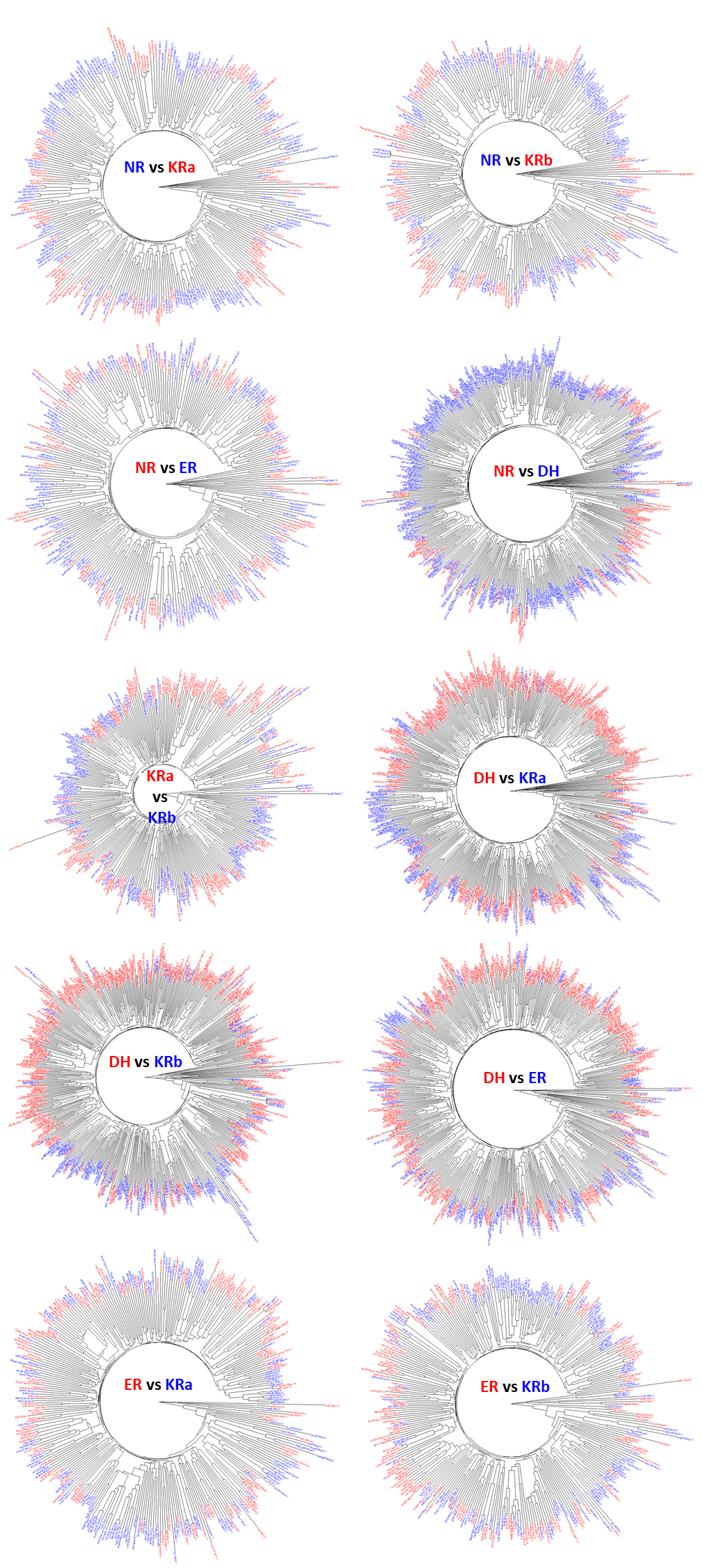


Figure S9: Phylogenetic trees for pairwise combinations of KS β-carbon type. KS sequences were aligned using MUSCLE^6, 7^. Aligned sequences were used to produce a pairwise evolutionary distance matrix using the maximum-likelihood distance estimation method^8^ in the Phangorn package^9^, using the WAG amino acid substitution model. Neighbour-joining trees were constructed from these distances and midpoint rooted.


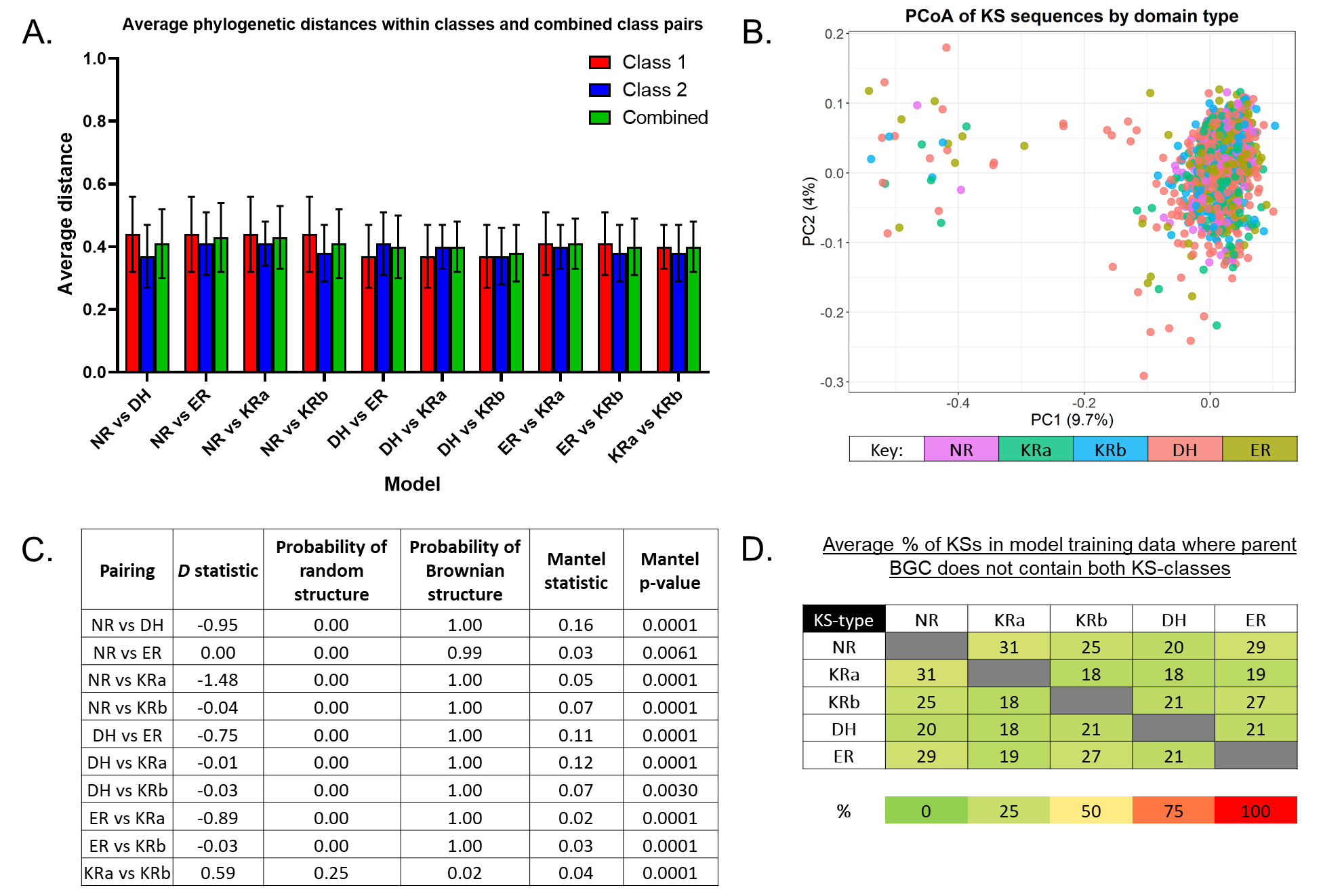


Figure S10: Phylogenetic analysis of KS sequences divisible by β-carbon type pairings. Analysis is performed on distance matrices described in figure S9 for panels A–C. **A.** Average pairwise evolutionary distances, divisible by β-carbon type or as a combined pairing. Grouping follows Class 1 vs Class 2 format. **B.** Principal Coordinate Analysis (PCoA) of total set of KSs. **C.** Fritz and Purvis *D* statistic^10^ and Mantel statistic^11, 12^ for β-carbon type pairings. **D.** Average percentage of KSs used as model training data where the parent BGC does not contain both KS-types used in the classifier.

Figure S10.A indicates little difference in the average pairwise evolutionary distances between classes (comparing blue and red bars). The PCoA analysis in panel B does not display clustering by KS-type. This indicates the structures are highly related and do not have a distinct global separation – this agrees with the origin BGC being the primary determinant of the phylogeny^13^.

Panel C tests clustering within the trees. The Fritz and Purvis *D* statistic^10^ measures phylogenetic signal strength in binary traits by measuring the probability of the trait (KS-class) distribution against random and Brownian tree distributions. A score of 0 is indicative of tree with traits populated by Brownian motion (i.e. clustering by trait) and a score of 1 a random tree (no significant clustering). Negative *D* scores here indicate greater than expected clumping of KS-types than a Brownian distribution within the tree, which are observed in all pairings, except for the KRa vs KRb pair. For the majority of the pairings, this indicates that neighbouring KSs in the trees are more likely to be of the same KS-type (i.e. they cluster). For the KRa vs KRb, the *D* score of 0.59 places the tree between random and Brownian, indicating a more dispersed tree with respect to KS-type. KRa vs KRb is the only pairing that shares the same structural domain composition within a module, so we find this result curious.

A limitation of the *D* statistic is that it describes the topology of the tree without explicitly considering the branch lengths. The Mantel test statistic used in panel C is a correlation coefficient indicating the strength and direction of the relationship between the evolutionary distance matrices and their KS-type – this tests the pairwise distances and their association to the types. Here a score of 0 indicates no clear relationship, a score of 1 a strong positive correlation, and a score of *–*1 a strong negative correlation. The Mantel scores show a weak but significant association between the KS-types, indicating that the sequences cluster but the overall sequence divergence is low.

Collectively, these analyses do not reveal anything new or surprising about c*is*-AT mPKS KSs – KS structures are highly similar, the substrate is not the primary phylogeny determinant, and β-carbon types are spread across the trees.

Given this information, we propose two non-exclusive strategies a binary classifier could take for learning to discriminate between KS-types:

1. Learn features of KSs that are generalizable to the substrate type (desirable).
2. Learn a series of cluster-specific features that correspond to the BGC origin and are independent of substrate specificity (undesirable).

For pathways that contain a mixture of KS-types used in the model, learning substrate-independent, BGC-specific features would be counterintuitive and expected to lead to test partition divergence (Figure 3). Panel D highlights that whilst strategy 2 will be counterintuitive for the majority of pathways, this will not be the case for *all* pathways, and so there is some inherent risk for the BGC-specific elements to influence model predictions. We would expect the influence of phylogeny to be idiosyncratic to clusters (i.e. poorly generalizable across the trees) and so this would be infrequently detected by the model explainer, translating to “tepid” results in the heat maps. An argument could be made to remove the possible influence of phylogeny by removing these sequences by manual curation; however, training data size and diversity is intrinsic to the performance of neural networks, and so there is an adverse trade-off for doing so. The pipeline we describe in the main article was built with directing mutagenesis in mind – we found that the parallelisable nature of these experiments provides enough risk tolerance for some failure (phylogeny-induced or otherwise), and so an inclusive model was preferable. Other researchers may feel otherwise, and scripts for training more strict models are available on our GitHub repository.


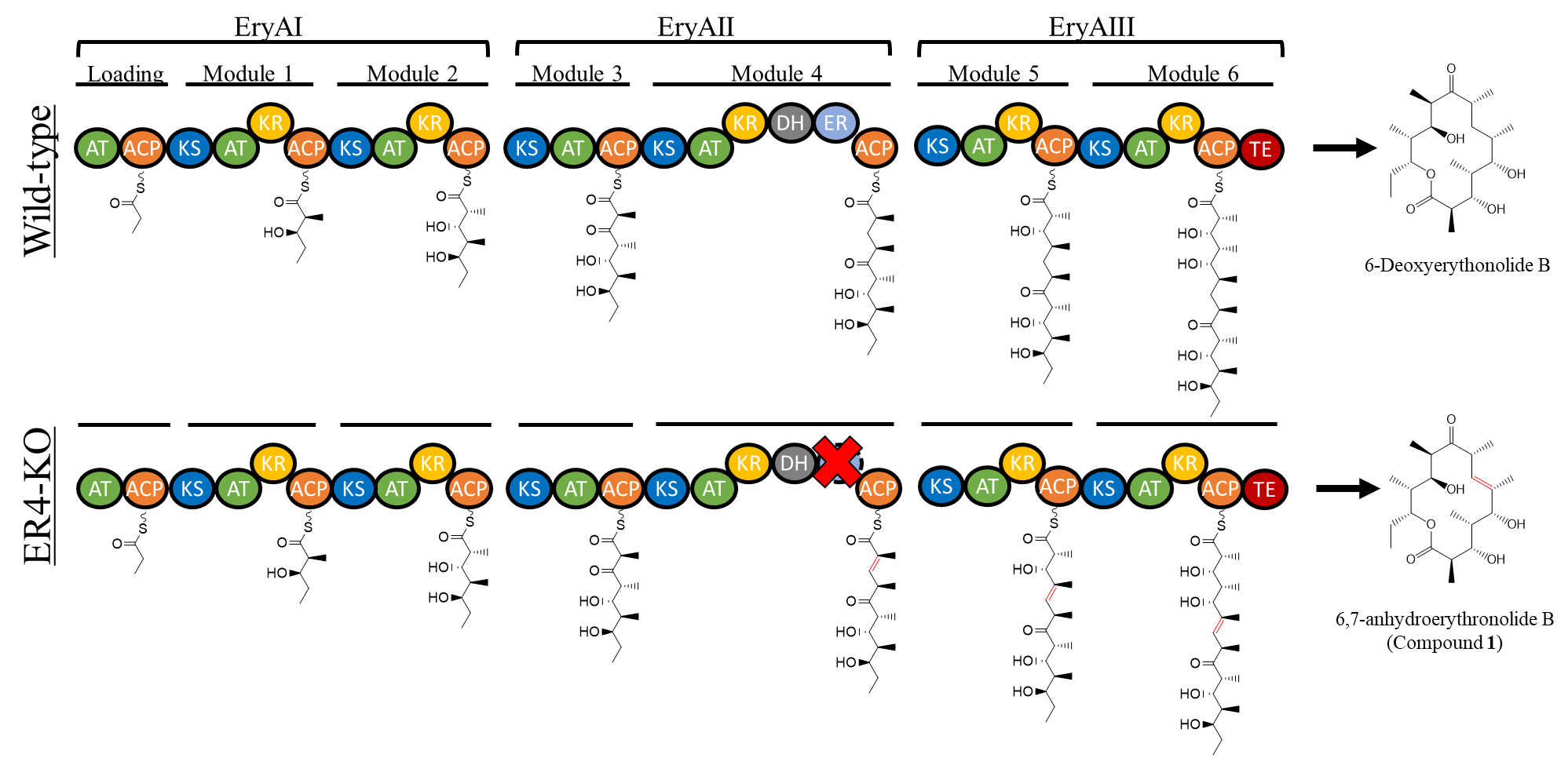


Figure S11: Polyketide synthase schematic for the biosynthesis of 6-deoxyerythonolide B by mPKS genes EryAI-III (top) and anticipated changes to synthesis brought about by the ER4 knockout described by Donadio et al. 1993 ^14^. The alkene group (red) is produced in module 4.

# Comparison of β-carbon binary classifier heat maps across models

Figures S12 and S13 display figures 5 and 6 with the regional overlay used as search areas explored in the DH vs ER *in vivo* mutagenesis experiment.

In all models, residues in region 1 (green) are detected, though there is inconsistency over which residues appear hot. As stated in the main text, we do not have a sequence-based explanation for why region 1 is considered important. The mutagenesis experiment that we ran found that both P1M and G6M resulted in near non-functional mPKSs. Structurally, this region immediately follows the docking domain, so it may be that the impact on the pathway was related to the docking function rather than the substrate.

Pocket A and B (magenta and purple) are consistently detected by in the models. Our best explanation detection here comes from the logo diagrams for this region (figures S12–16), where residues in pocket A in the VFxGxxxxxY motif have the best explanation of variance. Conventional sequence alignments at this position can be found in Hirsch et al. 2021^15^.

The gating loops (red) are detected in all models, though the logo diagrams do not give a strong narrative as to why.


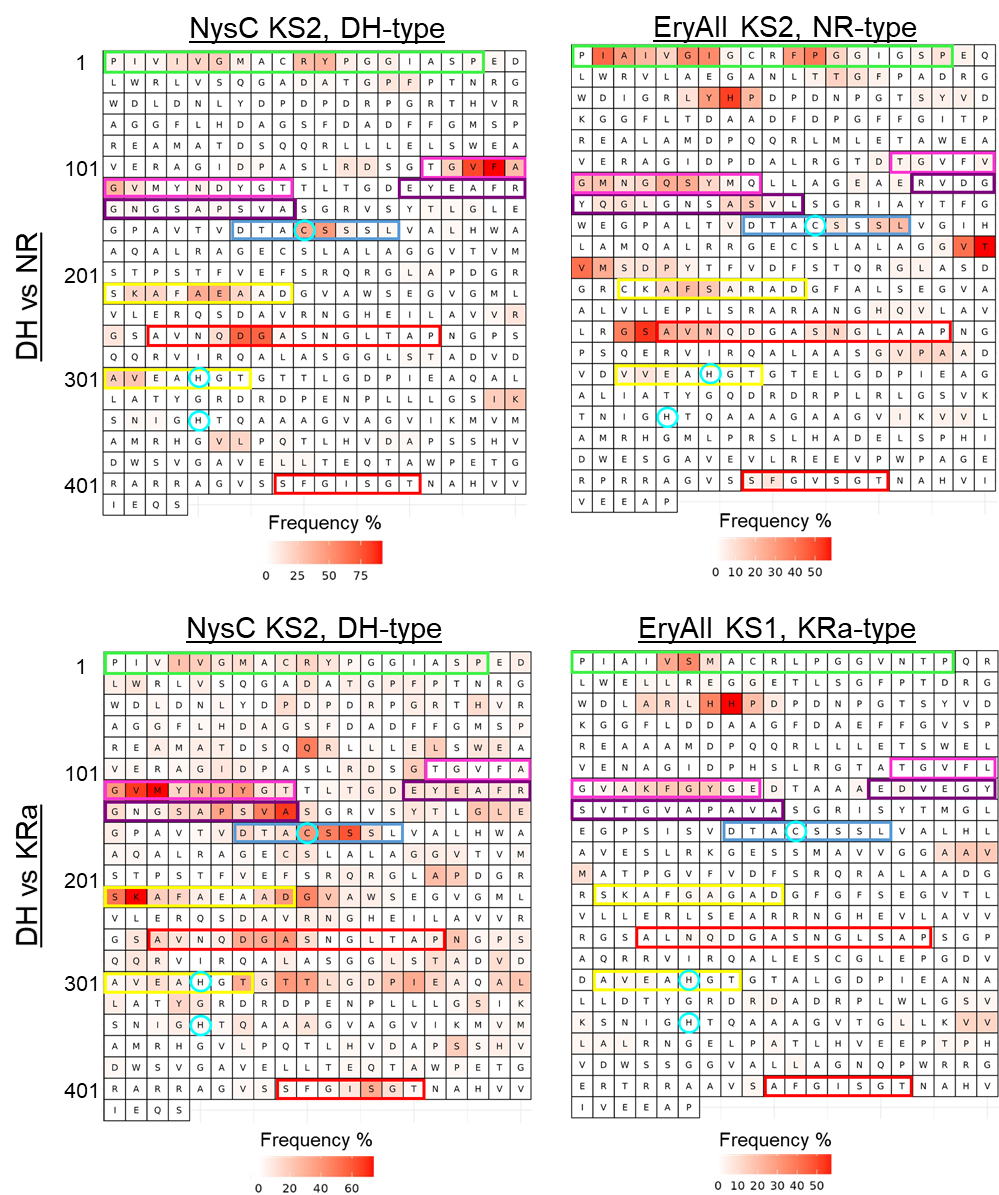

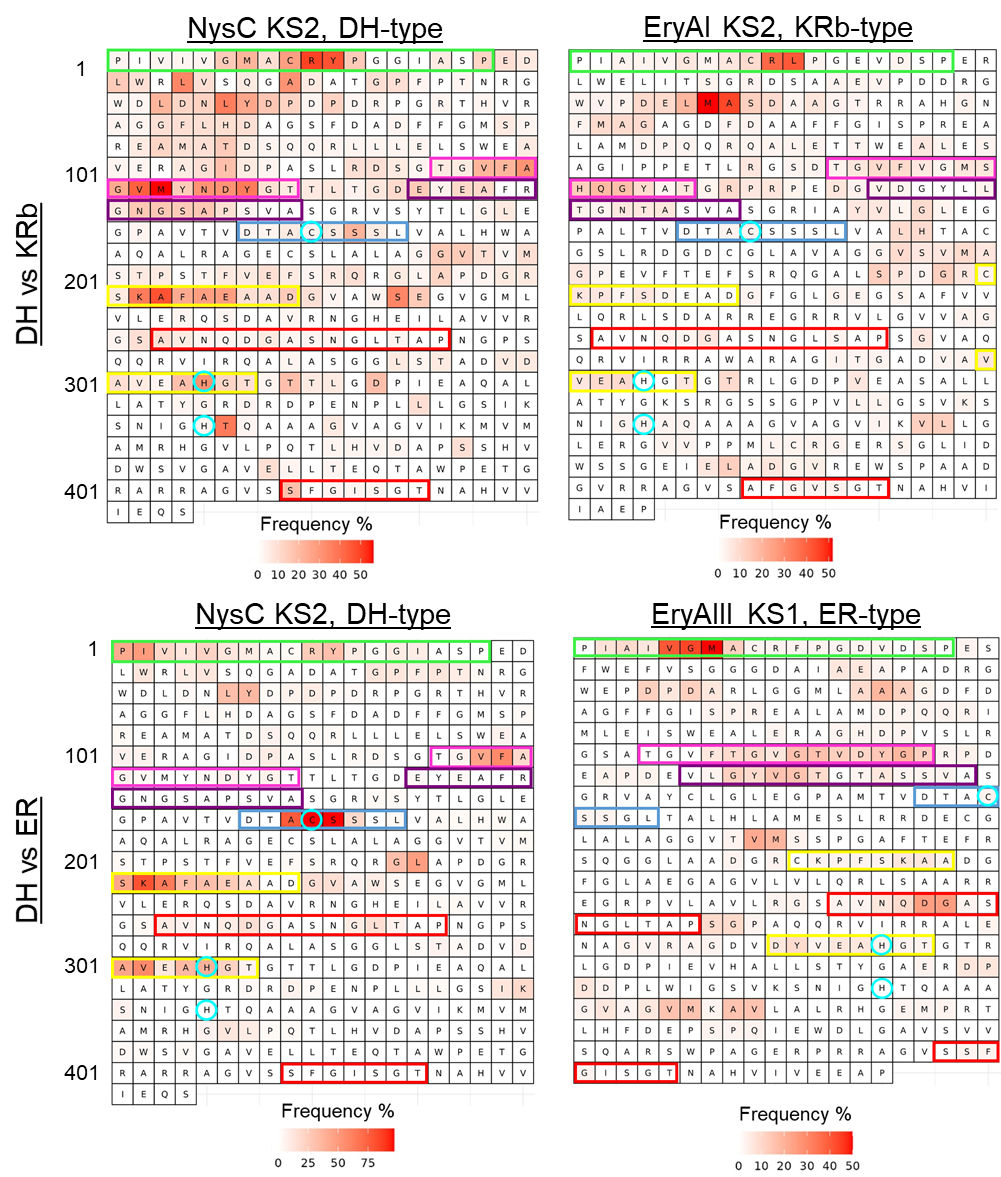


Figure S12: Aligned, monomeric explainer heat maps with regional overlay for models trained with DH-type KSs. Regions and colouring correspond to figures 7 and 8.


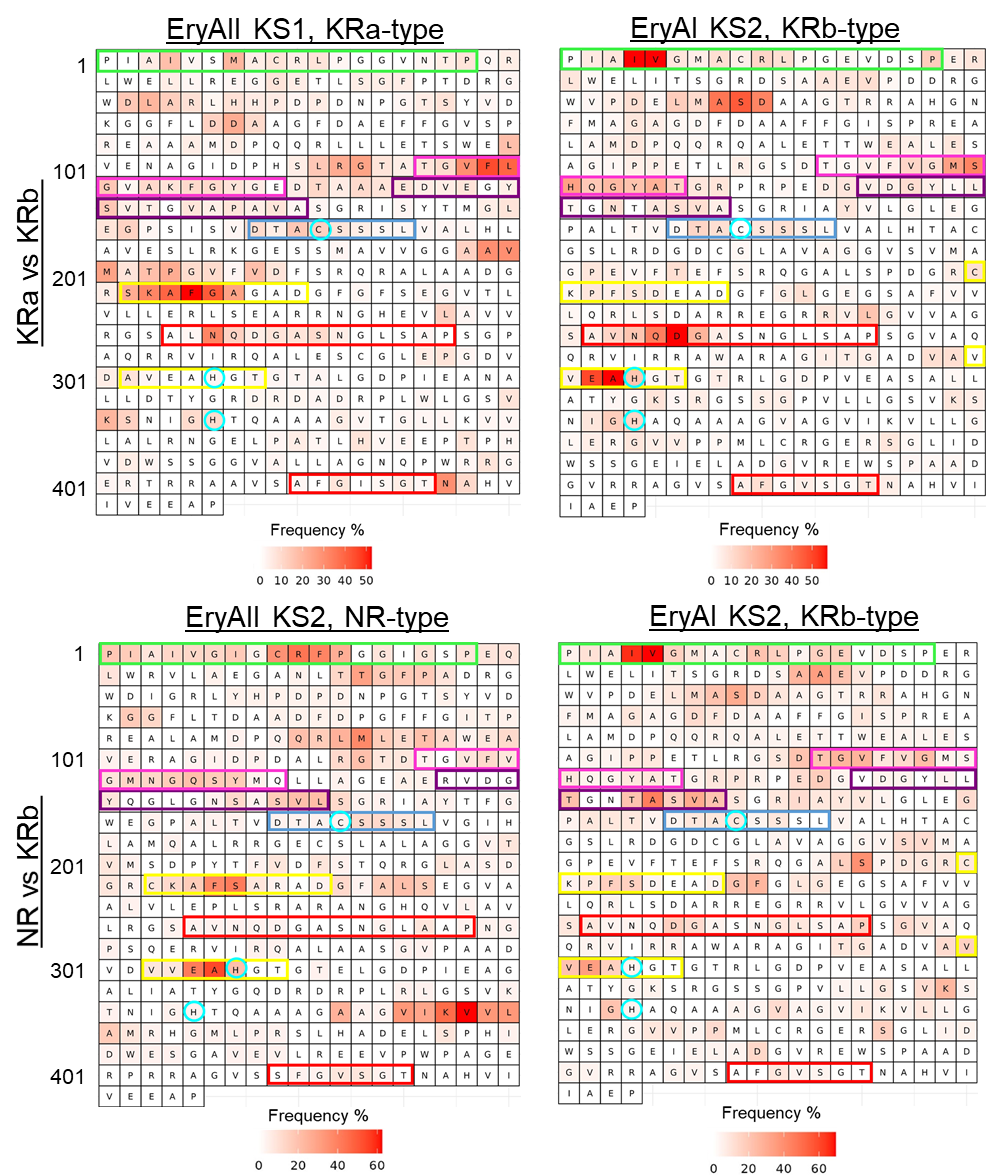


Figure S13: Aligned, monomeric explainer heat maps with regional overlay for models KRa vs KRb and NR vs KRb. Regions and colouring correspond to figures 7 and 8.


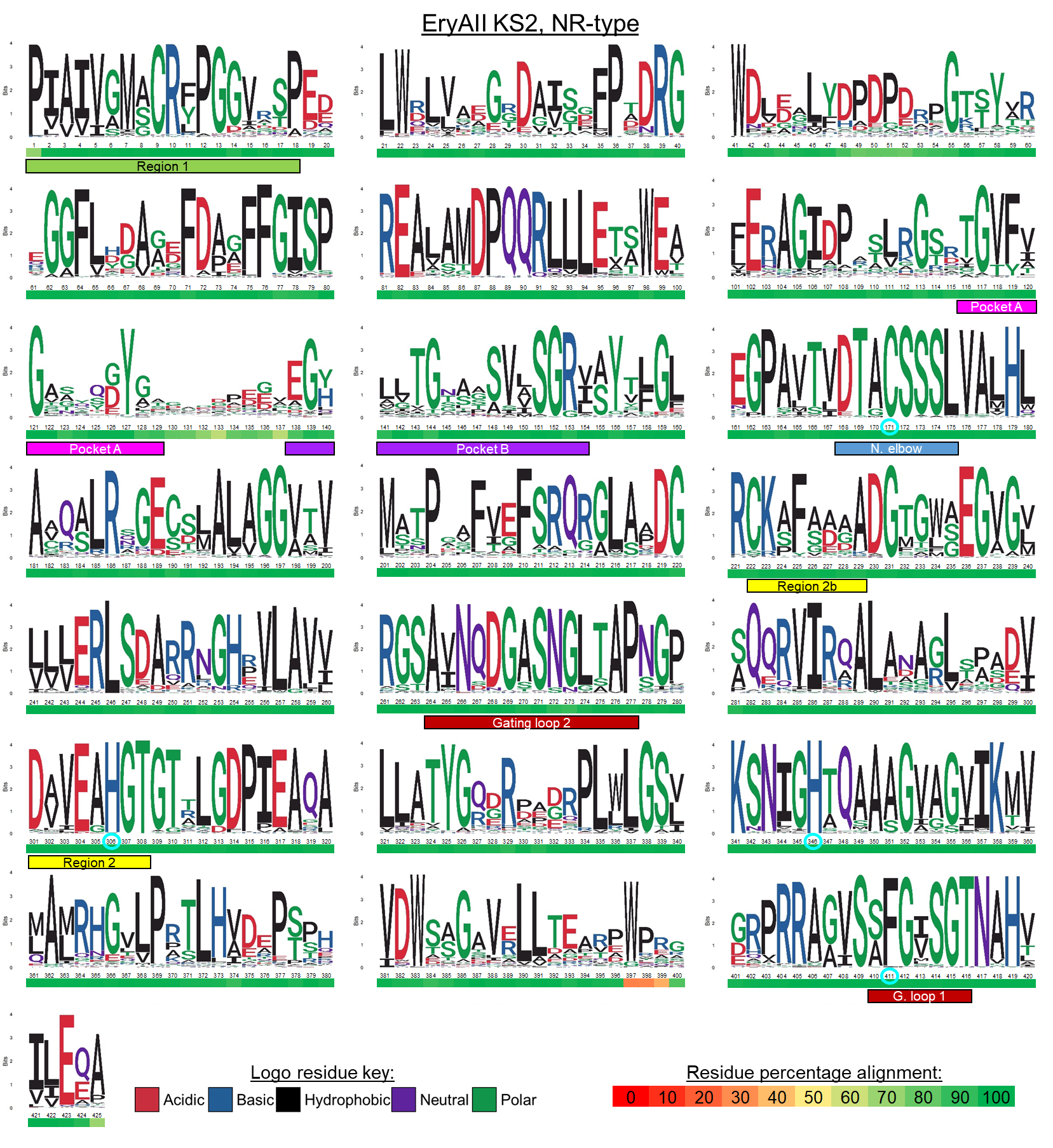


Figure S14: 2.5 Å sequence alignment of NR-type KSs to EryAII KS2.


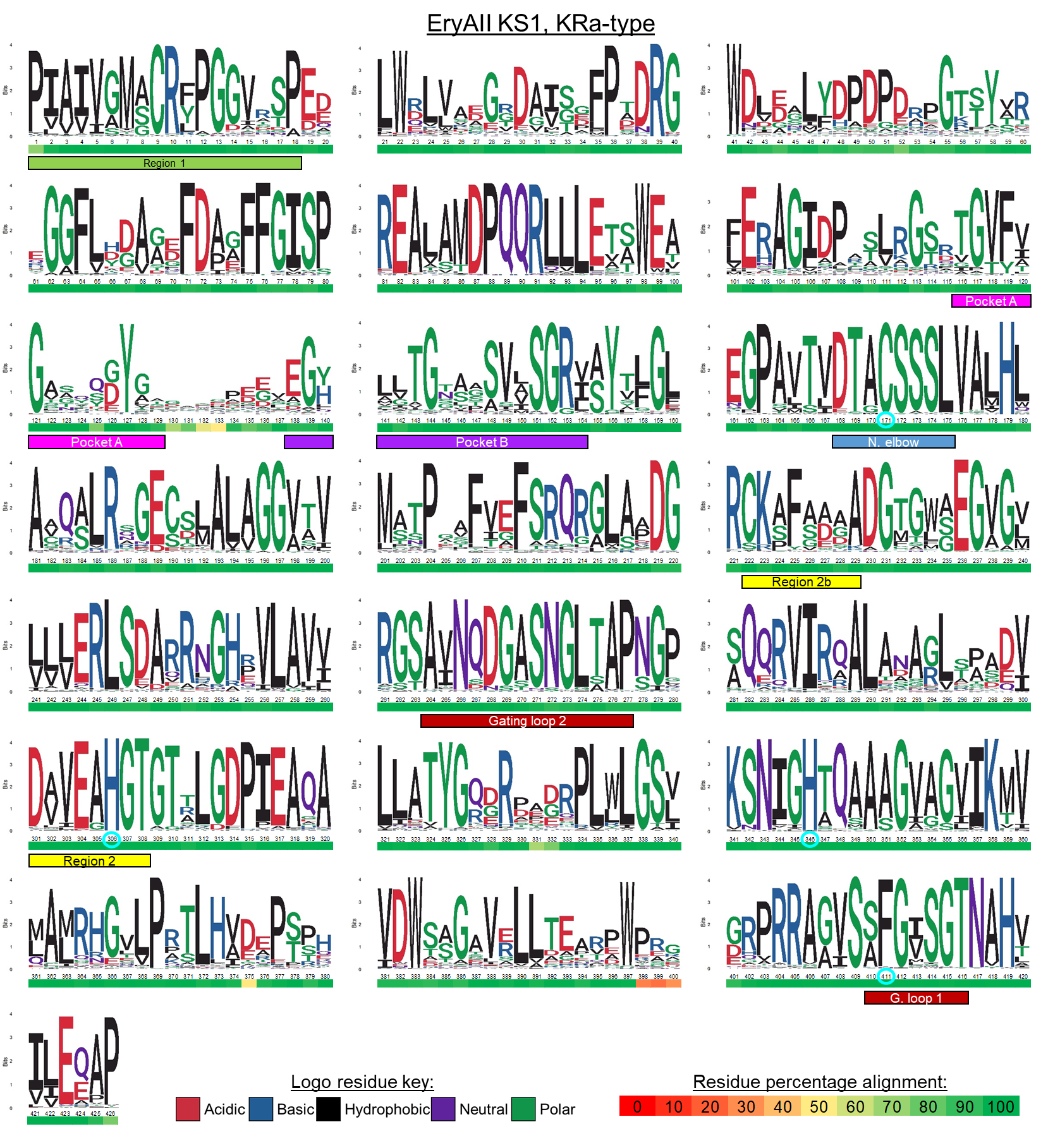


Figure S15: Logo diagrams produced from 2.5 Å structure alignment of KRa-type KSs to EryAII KS1.


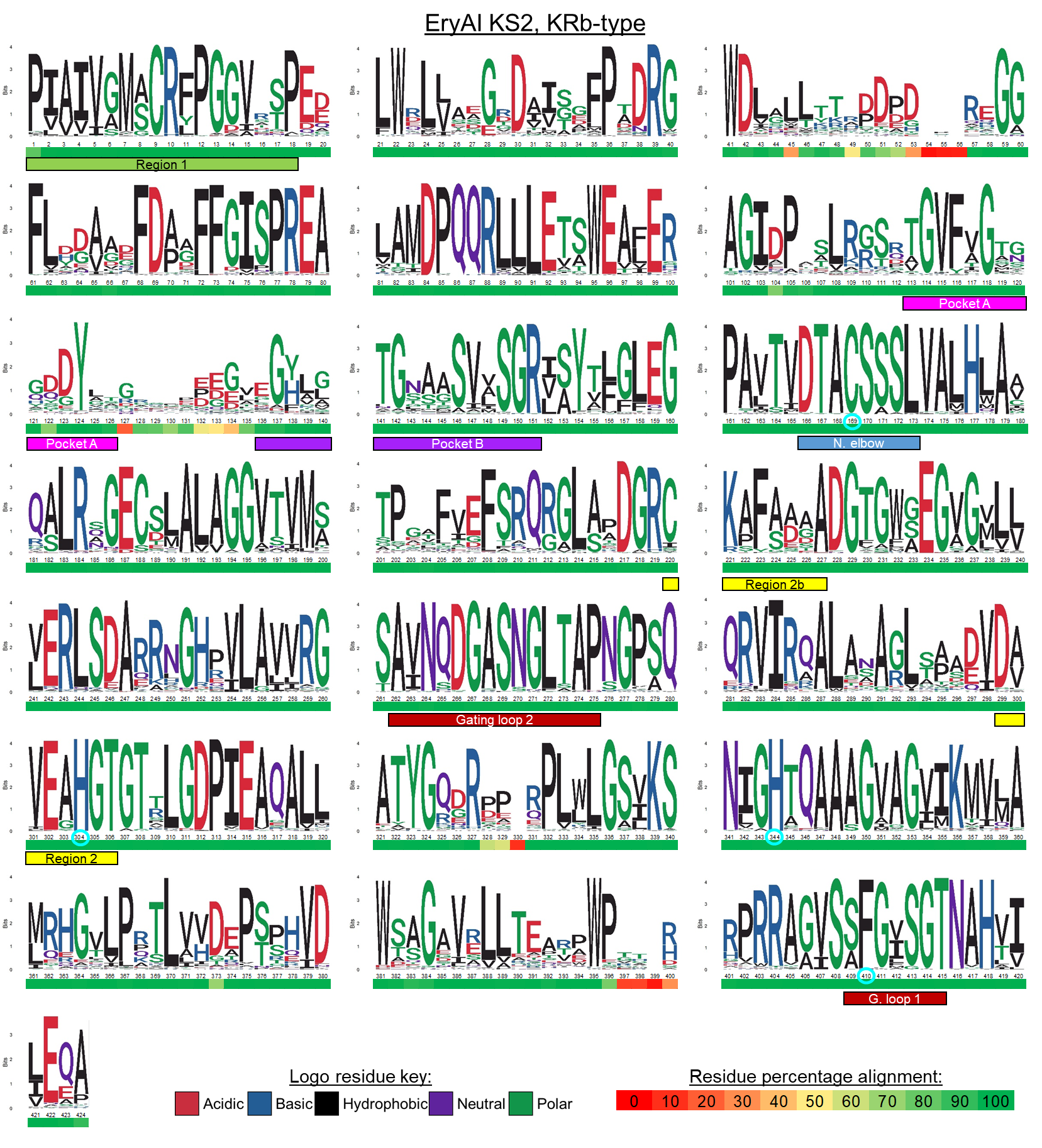


Figure S16: Logo diagrams produced from 2.5 Å structure alignment of KRb-type KSs to EryAI KS2.


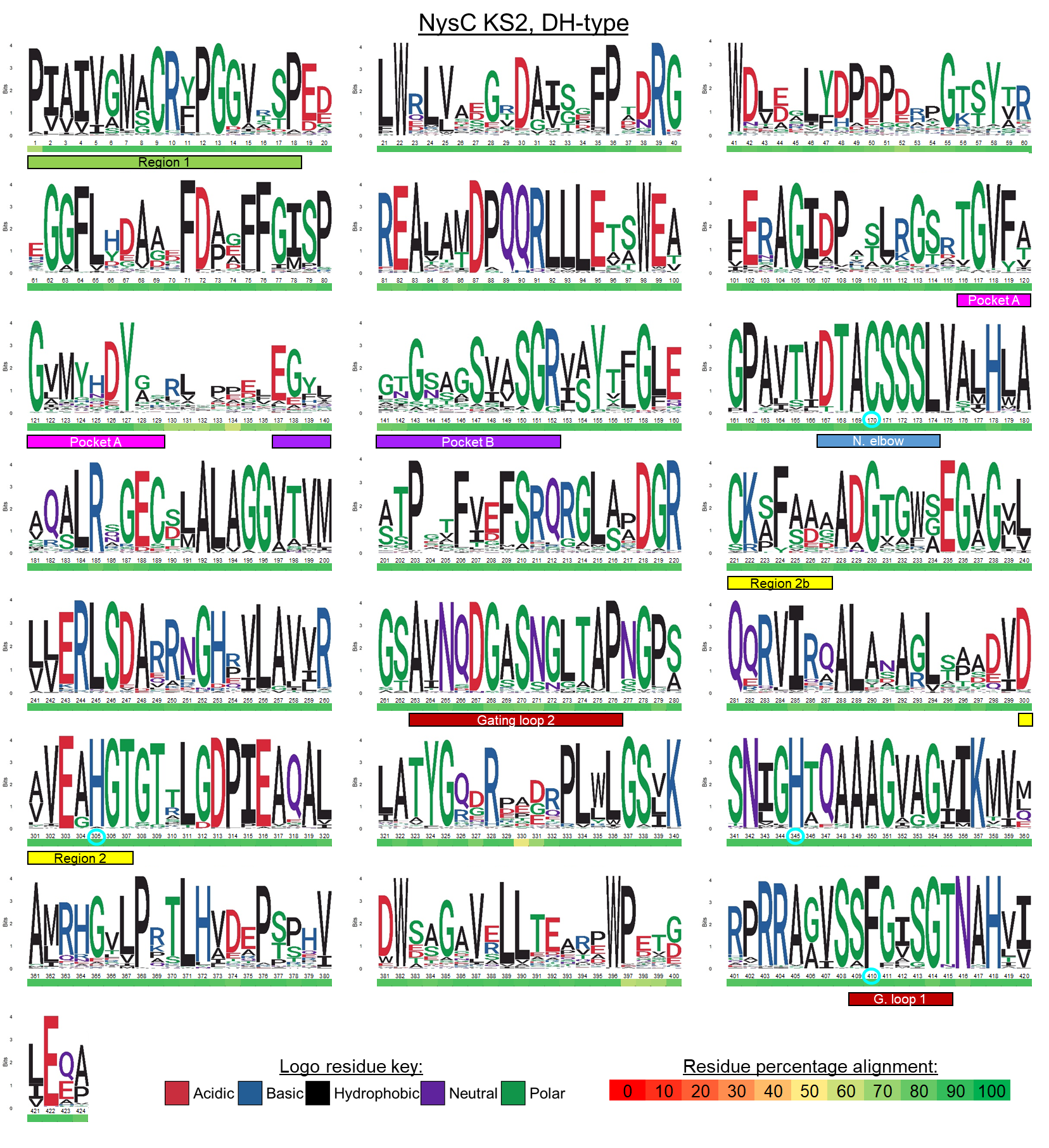


Figure S17: Logo diagrams produced from 2.5 Å structure alignment of DH-type KSs to NysC KS2.


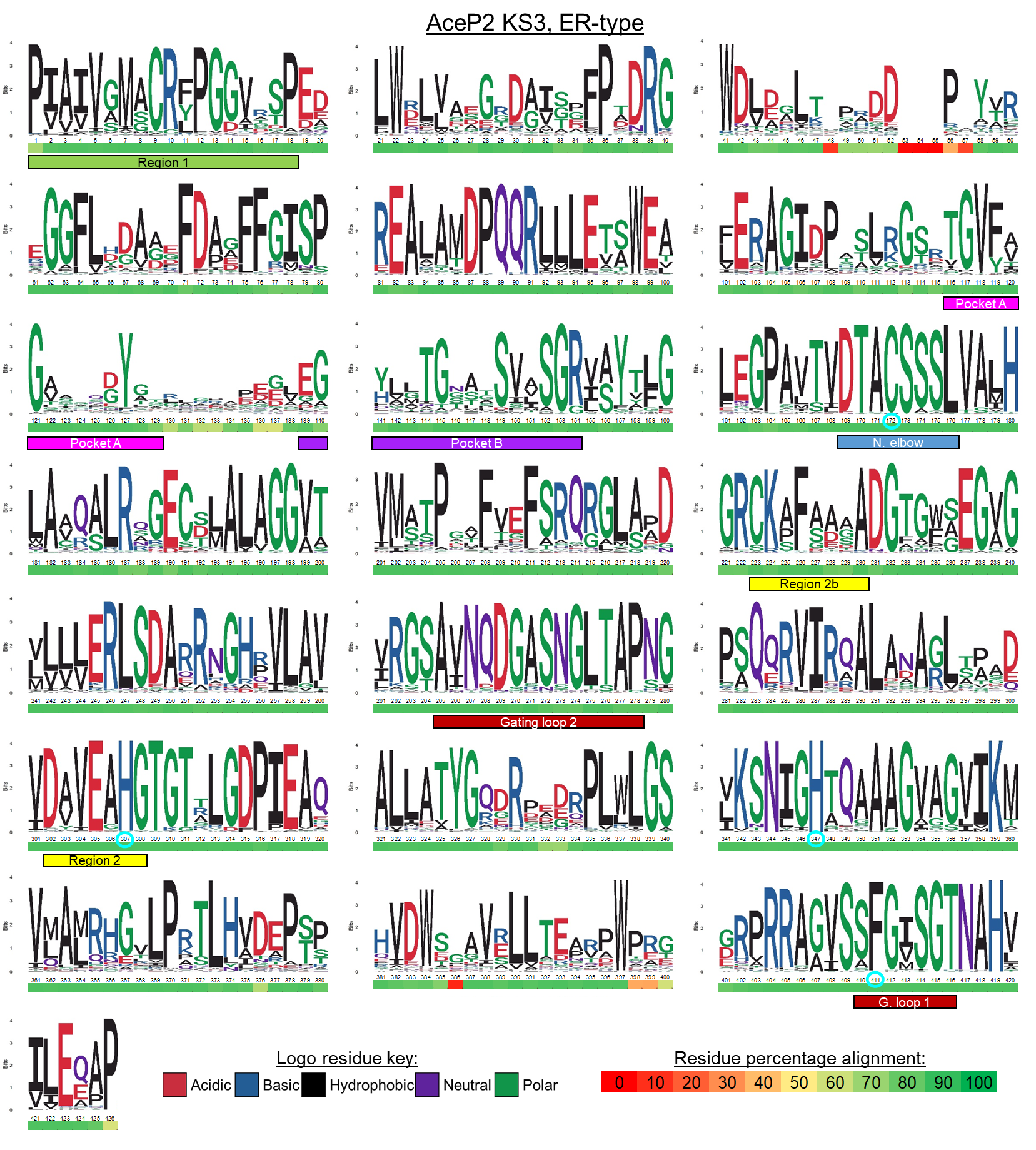


Figure S18: Logo diagrams produced from 2.5 Å structure alignment of ER-type KSs to AceP2 KS3.


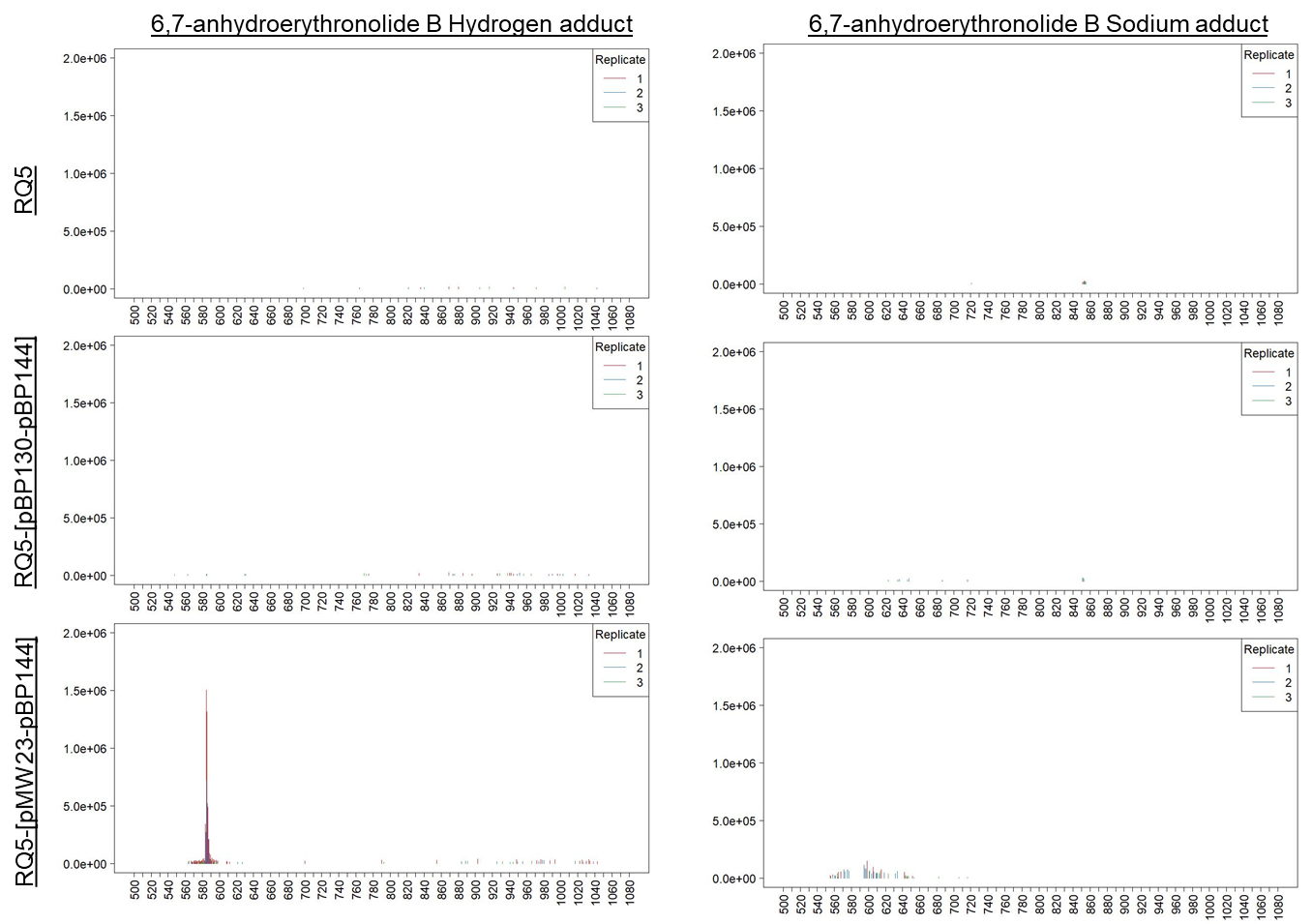


Figure S19: Extracted ion count intensity over time for 6,7-anhydroerythronolide B (compound **1**) hydrogen and sodium adducts with a 2 ppm filter. Hydrogen adduct mass range: 385.2577–385.2593. Sodium adduct mass range: 407.2402–407.2418. Rows correspond to strain: **Top:** RQ5 contains no DEBS expressing plasmids, **Middle:** pBP130 and pBP144 produce wild-type DEBSI-III, **Bottom:** plasmid pMW23 is a modified pBP130 containing the ER knockout mutation on module 4. These graphs indicate that compound **1** was only detecable in ER knockout strain.

We also tested these strains for masses that would be expected if the DH domain was being skipped in module 4, with a ketone (hydrogen adduct mass range: 401.2526–401.2542, sodium adduct mass range: 423.2346–423.2362) or an alcohol (hydrogen adduct mass range: 403.2683–403.2699, sodium adduct mass range: 425.2501–425.2519) being produced instead of an alkene (Figure S11). These masses were not detected.


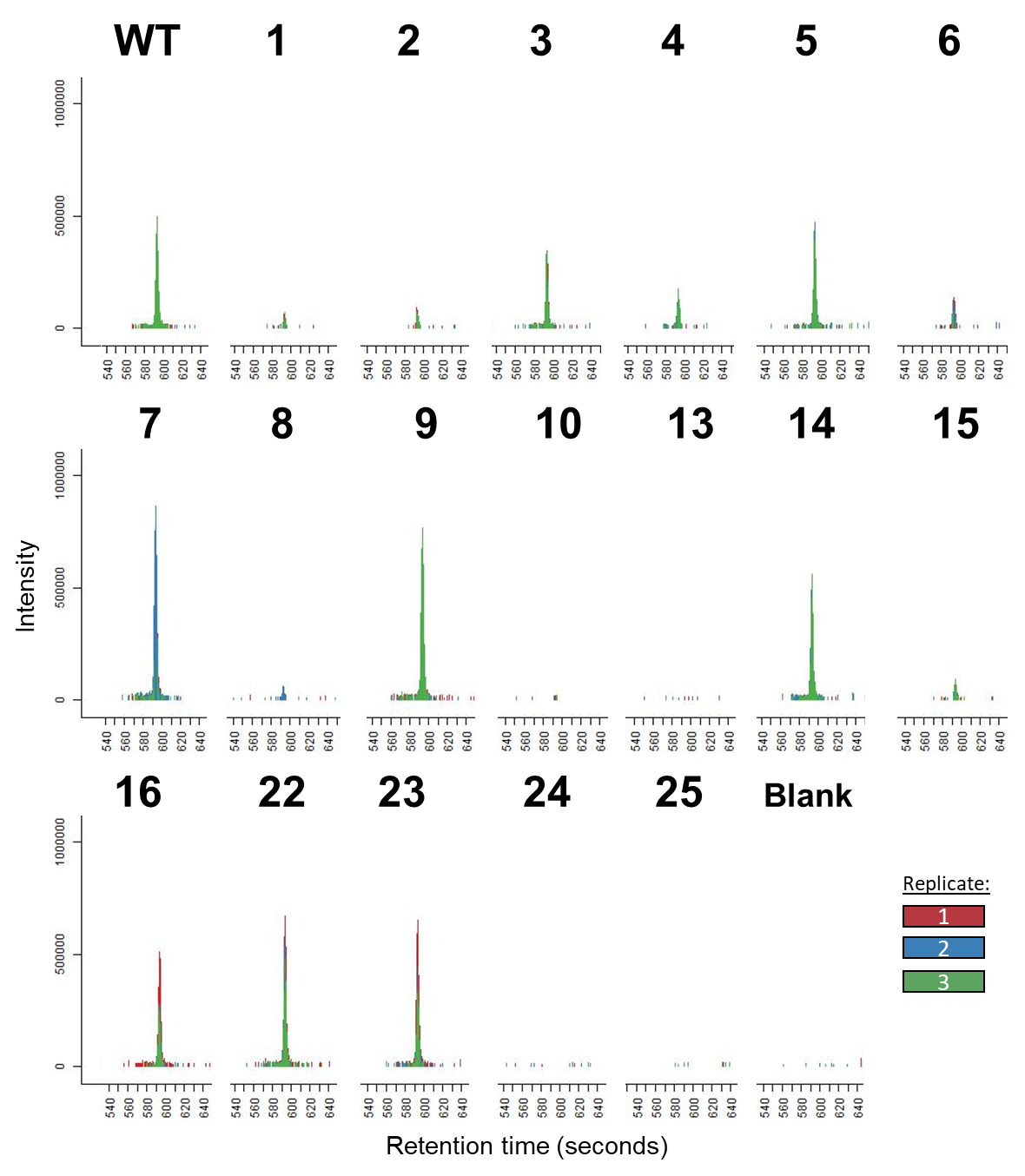
Figure S20: Extracted ion counts of hydrogen adduct of compound **1** (mass range 385.2577–385.2593) corresponding to mutant strains described in Figure 7.

Supplementary references

(1) Eng, C. H.; Backman, T. W. H.; Bailey, C. B.; Magnan, C.; Martin, H. G.; Katz, L.; Baldi, P.; Keasling, J. D. ClusterCAD: a computational platform for type I modular polyketide synthase design. *Nucleic Acids Research* **2018**, *46* (D1), D509-D515, Article. DOI: 10.1093/nar/gkx893.

(2) Jumper, J.; Evans, R.; Pritzel, A.; Green, T.; Figurnov, M.; Ronneberger, O.; Tunyasuvunakool, K.; Bates, R.; Žídek, A.; Potapenko, A.; et al. Highly accurate protein structure prediction with AlphaFold. *Nature* **2021**, *596* (7873), 583-589. DOI: 10.1038/s41586-021-03819-2.

(3) Cortes, J.; Haydock, S. F.; Roberts, G. A.; Bevitt, D. J.; Leadlay, P. F. An unusually large multifunctional polypeptide in the erythromycin-producing polyketide synthase of Saccharopolyspora erythraea. *Nature* **1990**, *348* (6297), 176-178. DOI: 10.1038/348176a0.

(4) Brautaset, T.; Sekurova, O. N.; Sletta, H.; Ellingsen, T. E.; Strøm, A. R.; Valla, S.; Zotchev, S. B. Biosynthesis of the polyene antifungal antibiotic nystatin in Streptomyces noursei ATCC 11455: analysis of the gene cluster and deduction of the biosynthetic pathway. *Chemistry & Biology* **2000**, *7* (6), 395-403. DOI: 10.1016/s1074-5521(00)00120-4.

(5) Sheehan, J.; Murphy, C. D.; Caffrey, P. New insights into polyene macrolide biosynthesis in Couchioplanes caeruleus. *Molecular BioSystems* **2017**, *13* (5), 866-873. DOI: 10.1039/c7mb00112f.

(6) Edgar, R. C. MUSCLE: multiple sequence alignment with high accuracy and high throughput. *Nucleic acids research.* **2004**, *32* (5), 1792-1797. DOI: 10.1093/nar/gkh340.

(7) Bodenhofer, U.; Bonatesta, E.; Horejš-Kainrath, C.; Hochreiter, S. msa: an R package for multiple sequence alignment. *Bioinformatics* **2015**, *31* (24), 3997-3999. DOI: 10.1093/bioinformatics/btv494.

(8) Guindon, S.; Gascuel, O.; Rannala, B. A Simple, Fast, and Accurate Algorithm to Estimate Large Phylogenies by Maximum Likelihood. *Systematic biology.* **2003**, *52* (5), 696-704. DOI: 10.1080/10635150390235520.

(9) Schliep, K. P. phangorn: phylogenetic analysis in R. *Bioinformatics.* **2011**, *27* (4), 592-593. DOI: 10.1093/bioinformatics/btq706.

(10) Fritz, S. A.; Purvis, A. Selectivity in Mammalian Extinction Risk and Threat Types: a New Measure of Phylogenetic Signal Strength in Binary Traits. *Conservation Biology* **2010**, *24* (4), 1042-1051. DOI: 10.1111/j.1523-1739.2010.01455.x.

(11) Oksanen, J.; Simpson, G. L.; Blanchet, F. G.; Kindt, R.; Legendre, P.; Minchin, P. R.; O'hara, R.; Solymos, P.; Stevens, M. H. H.; Szoecs, E. Vegan: community ecology package. **2001**.

(12) Legendre, P.; Legendre, L. *Numerical ecology*; Elsevier, 2012.

(13) Nguyen, T.; Ishida, K.; Jenke-Kodama, H.; Dittmann, E.; Gurgui, C.; Hochmuth, T.; Taudien, S.; Platzer, M.; Hertweck, C.; Piel, J. Exploiting the mosaic structure of trans-acyltransferase polyketide synthases for natural product discovery and pathway dissection. *Nature Biotechnology* **2008**, *26* (2), 225-233. DOI: 10.1038/nbt1379.

(14) Donadio, S.; Mcalpine, J. B.; Sheldon, P. J.; Jackson, M.; Katz, L. An erythromycin analog produced by reprogramming of polyketide synthesis. *Proceedings of the National Academy of Sciences* **1993**, *90* (15), 7119-7123. DOI: 10.1073/pnas.90.15.7119.

(15) Hirsch, M.; Fitzgerald, B. J.; Keatinge-Clay, A. T. How *cis*-Acyltransferase Assembly-Line Ketosynthases Gatekeep for Processed Polyketide Intermediates. *ACS Chemical Biology* **2021**, *16* (11), 2515-2526. DOI: 10.1021/acschembio.1c00598.
